# Characterization of KRAS^G12C^ inhibitor olomorasib single-agent and combination with activity in KRAS^G12C^-mutant models

**DOI:** 10.1038/s41467-026-72650-y

**Published:** 2026-05-04

**Authors:** Shengbin Peng, Youyan Zhang, Xi Lin, Chong Si, Robert Daniel Van Horn, Jack A. Dempsey, Eva Goetz, Robert J. Evans, Andrew Farber, Matthew J. Vandekopple, Wenyu Ming, Hong Gao, Chungping Yu, Wei Guo Xu, Nicholas E. Brown, Michele S. Dowless, Nicholas Pulliam, David A. Barda, Deqi Guo, Serge L. Boulet, Lysian Huber, Andrew Capen, Bonita Jones, Sarah Bogner, Mark A. Castanares, Jennifer Rachelle Stephens, Megan A. Johnson, Carmen L. Curtis, John M. Strelow, Junpeng Xiao, Josh Ballard, Wayne P. Bocchinfuso, Michael J. Chalmers, Jing Wang, Jorg Hendle, Melbert D. Saflor, Danalyn Manglicmot Lagutan, Tarun Gheyi, Anita Sarkar, Margaret Kearins, Frances Tung, Joseph Ho, Logan Rodgers, Jordi Benach, Anton Joseph Frommelt, Lian Zhou, Bradley L. Ackermann, Denis McCann, Anke Klippel, Sean G. Buchanan, James R. Henry, Xueqian Gong

**Affiliations:** Eli Lilly and Company, Indianapolis, IN USA

**Keywords:** Oncogenes, Cancer, Drug development

## Abstract

The impact of first-generation covalent KRAS^G12C^ inhibitors has been reduced due to the development of drug resistance, tolerability and challenges combining with immunotherapy. We designed olomorasib, a next-generation GDP-binding KRAS^G12C^ inhibitor, for nanomolar potency as well as selectivity over wild-type inhibition. In both in vitro and in vivo models of KRAS^G12C^ -mutant cancers, olomorasib reduces RAS activity and pERK levels, leading to substantial and significant tumor growth inhibition. Additionally, olomorasib combined with immune checkpoint inhibitors demonstrates greater anti-tumor activity compared to monotherapy. Furthermore, we demonstrate that olomorasib binds tightly to *KRAS*^*G12C*^ even in the presence of clinically relevant second site mutations, a known mechanism of resistance and limitation to currently approved *KRAS*^*G12C*^ inhibitors. These findings suggest that olomorasib could be effective for patients with *KRAS*^G12C^ mutant cancers either as monotherapy or in combination with immunotherapy. Olomorasib monotherapy and combination treatments are currently being investigated clinically.

## Abstract (web summary)

The success of KRAS G12C mutation specific inhibitors in patients with KRAS-driven tumours is limited by the emergence of acquired resistance. Here, the authors characterise olomorasib, a next-generation covalent KRAS G12C-mutant inhibitor, demonstrating efficacy in the presence of clinically relevant resistance mutations in preclinical KRAS-driven cancer models.

## Introduction

The KRAS GTPase is the most frequently mutated oncogene in cancer and was once considered an undruggable target due to its smooth and shallow surface as well as high affinity and avidity for intracellular guanosine nucleotides (GTP and GDP)^1,2^. KRAS mutations are diverse, with the most common amino acid substitutions occurring at G12, G13, and Q61, resulting in increased KRAS activity and constitutive activation of downstream signaling^3,4^. Of these oncogenic mutations, KRAS^G12C^ occurs in nearly 13% of lung adenocarcinomas, 4% of colorectal cancers, and at lower rates in other cancer types^5^. Clinically, KRAS mutations are associated with poor patient prognosis and decreased response to standard of care therapies^6^.

Given the clinical relevance of KRAS mutations, there have been intense efforts to target KRAS as well as downstream signaling components, which were met with limited success^2,6^. A recent breakthrough in medicinal chemistry led to the discovery of small-molecule inhibitors that covalently bind KRAS^G12C^, within the switch II pocket region^7^, trapping the protein in the inactive (GDP-bound) state and inhibiting KRAS-mediated signaling. Currently, there are two FDA-approved KRAS^G12C^ inhibitors for patients previously treated with standard of care (SOC) and with KRAS^G12C^ mutated non-small cell lung cancer (NSCLC): adagrasib^8^ and sotorasib^1^. Both drugs have improved outcomes in KRAS^G12C^ mutated NSCLC, though efficacy is limited by adverse events resulting in dose modifications (reduction or interruption) and discontinuation of treatment, as well as the emergence of acquired resistance^9^, diminishing overall target engagement and pathway inhibition^10,11^.

The most frequently observed adverse events of adagrasib and sotorasib were diarrhea, nausea, fatigue and liver toxicity. Due to adverse events of high-grade liver toxicity, studies have demonstrated limited ability to combine sotorasib with NSCLC standard of care PD-L1 inhibitor pembrolizumab^12,13^, and adagrasib required frequent dose modifications when used in combination with pembrolizumab^9^. Moreover, emerging clinical data revealed multiple mechanisms of resistance in patients treated with adagrasib and sotorasib, including secondary KRAS mutations, activation of bypass pathways, and upregulation of receptor tyrosine kinases (RTKs)^14–16^. Understanding the resistance mechanisms is crucial for developing strategies to overcome or prolong the onset of resistance and improve patient outcomes.

Due to the ubiquitous nature of RAS signaling in normally functioning cells, the pathway is tightly regulated to ensure proper control of cell growth, proliferation, and survival^17,18^. Upstream signals from RTKs flow through guanosine nucleotide exchange factors (GEFs) such as SOS1 to activate KRAS and the RAF-MEK-ERK signaling cascade. Several compensatory pathways have also been identified to regulate this signaling network^10^. These mechanisms highlight the potential for combination strategies^5,19,20^ to more effectively target KRAS^G12C^ mutant cancers and suppress RAS-mediated signaling, leading to increased anti-tumor activity. To this point, signaling constituents currently under preclinical or clinical investigation in combination with KRAS inhibitors include: EGFR^21^, ERK1/2^10^, SOS1^22^, SHP2^23^, CDK4/6^10,24^, PI3K^25^ and PD-L1^26^. As observed in studies of adagrasib and sotorasib, toxicity may limit clinical benefit of these combination strategies^11^, and novel KRAS^G12C^ inhibitors should be amenable to combination strategies.

Here, we characterize LY3537982 (hereafter olomorasib), a highly potent and selective KRAS^G12C^ inhibitor, in biochemical and cellular studies, and in xenograft and patient-derived xenograft (PDX) efficacy studies, both alone and in combination with other inhibitors. Additionally, we explore the activity of olomorasib in the context of second site KRAS^G12C^ mutations, which may limit response to current clinically available KRAS^G12C^ inhibitors. Finally, we investigate mechanisms which confer resistance to KRAS^G12C^ inhibitors and treatments that can potentially overcome resistance. Olomorasib monotherapy and combination treatments are currently being investigated clinically (NCT04956640 and NCT06119581).

## Results

### Biochemical and in vitro characterization of Olomorasib

**Fig. 1:**
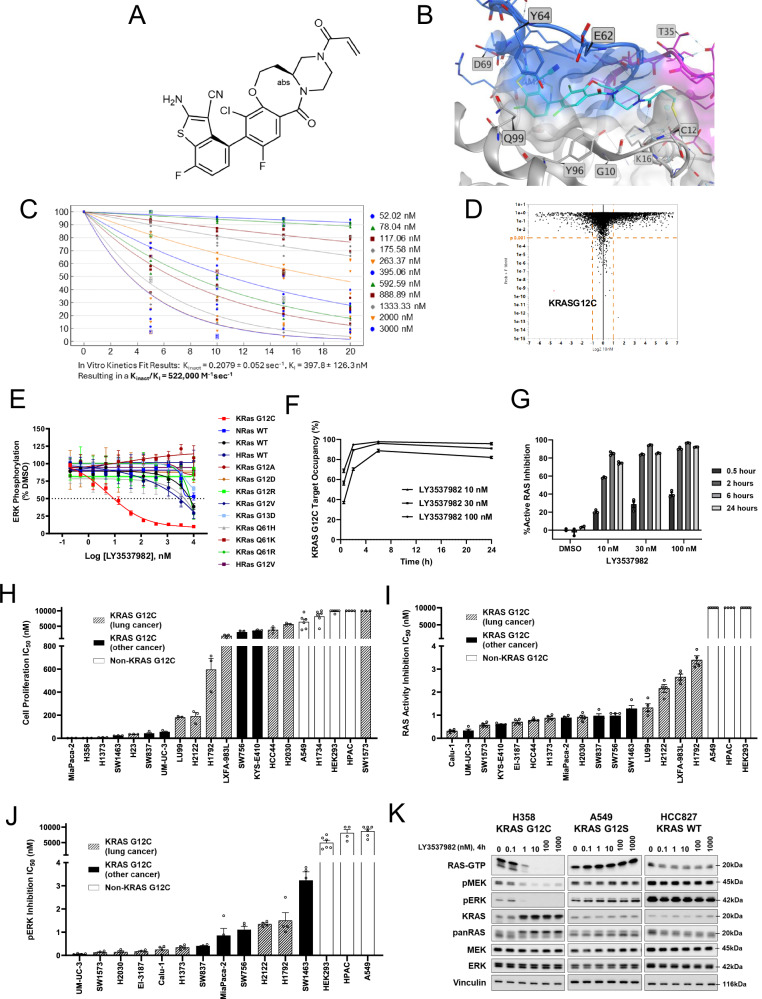
Biochemical and in vitro characterization of Olomorasib. **A** Structure of olomorasib. **B** Co-crystal structure of olomorasib with KRAS^G12C^. The blue mesh represents the 2Fo–Fc electron density map contoured at 1.0σ. **C** Olomorasib binding kinetics for KRAS^G12C^. **D** Olomorasib selectivity and specificity for KRAS G12C by use of cysteine probe (*n* = 2 experimental replicates, jPOST repository: PXD070323) and **E** ERK phosphorylation assay (*n* = 3 biological replicates). **F** Olomorasib target occupancy at the indicated concentration of compound (*n* = 3 biological replicates). **G** Active RAS inhibition by olomorasib at the indicated concentration and time (Data represent 3 (treated) or 2 (DMSO) independent experiments, mean ± SEM). A panel of cancer cells were treated with olomorasib and **H** absolute IC_50_ values (*n* = 3-6 biological replicates) **I** RAS activity inhibition (*n* = 3-6 biological replicates) and **J** pERK inhibition was determined. Cell line identity and KRAS^G12C^ mutation status are indicated in the figure (*n* = 4 biological replicates). **K** Western blot analysis against the indicated antibodies in cell lines of varied KRAS mutations and status was performed. Cells were treated for 24 h unless indicated otherwise. Source data are available. Data are presented as mean ± SEM.

A structure-based drug design approach, including optimization for favorable drug-like properties based on a previous internal KRAS^G12C^ effort (patent: WO2020081282; ref. ^27^, Supplementary Note), led to the development of olomorasib, as a potent and selective covalent GDP-binding KRAS^G12C^ inhibitor (Fig. 1A). Utilizing a custom pET26b *Escherichia coli* expression vector in-frame with a N-terminal hexahistidine affinity tag we generated an olomorasib-bound crystal structure of KRAS^G12C^ of 1.14 Å (Fig. 1B, Supplementary Fig. 1A, Supplementary Table 1). We determined the structure by molecular replacement with a model derived from the structures of a KRAS-G12C with GDP (4L8G^7^). The final refined model reveals that the acrylamide group of the ligand (warhead) can form a covalent bond with cysteine 12 and extend into the switch II region (blue), while the benzothiophene group and surrounding amino acids form a strong van der Waals interaction. Additionally, the benzothiophene group formed a direct hydrogen interaction with D69 and E63. Importantly, neither H95 nor Y96 directly hydrogen bonded with the molecule but had hydrophobic interactions with the ligand.

The structural alignment of the inactive form of KRAS^G12C^ with covalently bound olomorasib (gray, with SW-I in pink and SW-II in deep blue) and the active form of KRAS^G12C^ (orange, with SW-II partially visible due to its flexibility) in complex with GNP and CRAF (blue) (PDB ID: 6XHB^28^) reveals significant conformational changes (Supplementary Fig. 1B). Upon covalent binding to cysteine 12, olomorasib locks the SW-I/SW-II regions into an inactive conformation, thereby blocking interaction with CRAF. This binding induces the formation of a salt bridge between R68 of the SW-II loop and E37, which are critical residues for interaction with R59/R67/CRAF. Additionally, conformational changes in E31 and D33 further obstruct the interaction with K84/CRAF.

The olomorasib-mediated rate of hydrolysis (k_hy_) of RAS-GTP to RAS-GDP was 2.2 h^-1^ and the rate of exchange (k_ex_) of RAS-GDP to RAS-GTP was 6.2 h^-1^. The k_inact_/K_I_ of olomorasib engagement of RAS-GDP was 522,000 M^-1^sec^-1^ (Fig. 1C) compared to 35,000 M^-1^sec^-1^ for adagrasib^29^ and 9900 M^-1^sec^-1^ for sotorasib^30^. To determine the selectivity of olomorasib towards proteome-wide cysteines in vitro, a mass spectrometry-based competitive chemical proteomics study was performed. KRAS^G12C^ mutant H358 cells were treated with either olomorasib or DMSO, and incubated with isotope-labeled thiol-reactive olomorasib probes. The probe-labeled peptides were enriched and analyzed by LC-MS/MS^31^. Potent and statistically significant competition of olomorasib (at 10 nM, *p* < 0.05) with the cysteine probe was only observed for the peptide corresponding to KRAS^G12C^ (LVVVGACGVGK) (Fig. 1D), indicating that olomorasib is highly selective for KRAS^G12C^. To evaluate the potency and selectivity of olomorasib in cellular assays, ERK phosphorylation (pERK) in mouse embryonic fibroblast (MEF) RASless cells^32,33^ expressing human wild-type KRAS, KRAS^G12C^, and non-KRAS^G12C^-mutant forms of RAS was measured. In cells expressing wild type isoforms of RAS (K/N/HRAS) and most mutant forms of KRAS, olomorasib did not significantly affect ERK phosphorylation (Fig. 1E). However, in cells harboring KRAS^G12C^ we observed substantially decreased ERK phosphorylation, further demonstrating selectivity of olomorasib towards G12C mutant forms of RAS (Fig. 1E). To determine the efficiency of olomorasib KRAS^G12C^ binding and efficacy of RAS inhibition in cancer cells, KRAS^G12C^ target occupancy and RAS activity were assessed in SW1463 cells following compound treatment. We observed a dose-dependent increase in target occupancy (range: 35%-70%) within 30 min, and this was further increased to between 70%-95% within 2 h of treatment with olomorasib (Fig. 1F). RAS activity (RAS-GTP) was similarly and substantially decreased in a dose and time-dependent manner (Fig. 1G).

We next examined the effect of olomorasib on cell proliferation, active RAS levels and pERK inhibition in a panel of cancer cell lines, harboring either KRAS^G12C^, non-KRAS^G12C^ mutations or wild-type KRAS (Fig. 1H-J). Cells were treated with increasing concentrations of olomorasib and IC_50_ values for cell proliferation, RAS activity and pERK inhibition were determined for each cell line. Seven of 16 KRAS^G12C^-mutant cell lines tested showed sensitivity (IC_50_ < 100 nM) while all KRAS non-G12C-mutant cell lines were resistant to olomorasib (Fig. 1H). Additionally, olomorasib potently inhibited RAS activity (Fig. 1I) and suppressed pERK levels (Fig. 1J) in all KRAS^G12C^ cell lines tested. In contrast, in KRAS non-G12C-mutant cells active RAS and pERK levels were unaffected by the addition of olomorasib (IC_50_ > 1 µM). We next examined the effect of olomorasib on key KRAS pathway constituents in olomorasib-sensitive cell lines. H358 and MiaPaca-2 cells were treated with olomorasib (10 nM, 100 nM, or 1 µM) or DMSO. Post-treatment gene and protein expression of known RAS-regulated genes were measured. In both H358 and MiaPaca-2 cells, all RAS-regulated genes examined showed decreased expression relative to control, confirming down regulation of RAS-mediated gene expression by olomorasib in these models (Supplementary Fig. 1C). By Western blot analysis, pERK, pMEK, and RAS-GTP were decreased in a dose-dependent manner in KRAS^G12C^ mutant H358 and MiaPaca-2 cell lines following olomorasib treatment (Fig. 1K, Supplementary Fig. 1D). In cells expressing non-KRAS^G12C^ (G12S or WT) these effects were not observed (Fig. 1K). Furthermore, pS6 and pRb protein expression were similarly decreased in KRAS^G12C^ mutant cells, while cleaved PARP (cPARP) was increased in a dose and time-dependent manner, in the models examined (Supplementary Fig. 1D). Together, these data demonstrate the selectivity and potency of olomorasib towards KRAS^G12C^ and its activity in KRAS^G12C^-mutant cell lines, while sparing KRAS wild-type and non-KRAS^G12C^-mutant isoforms.

### Olomorasib in combination with additional anti-cancer therapeutics decreases cell viability in vitro

**Fig. 2:**
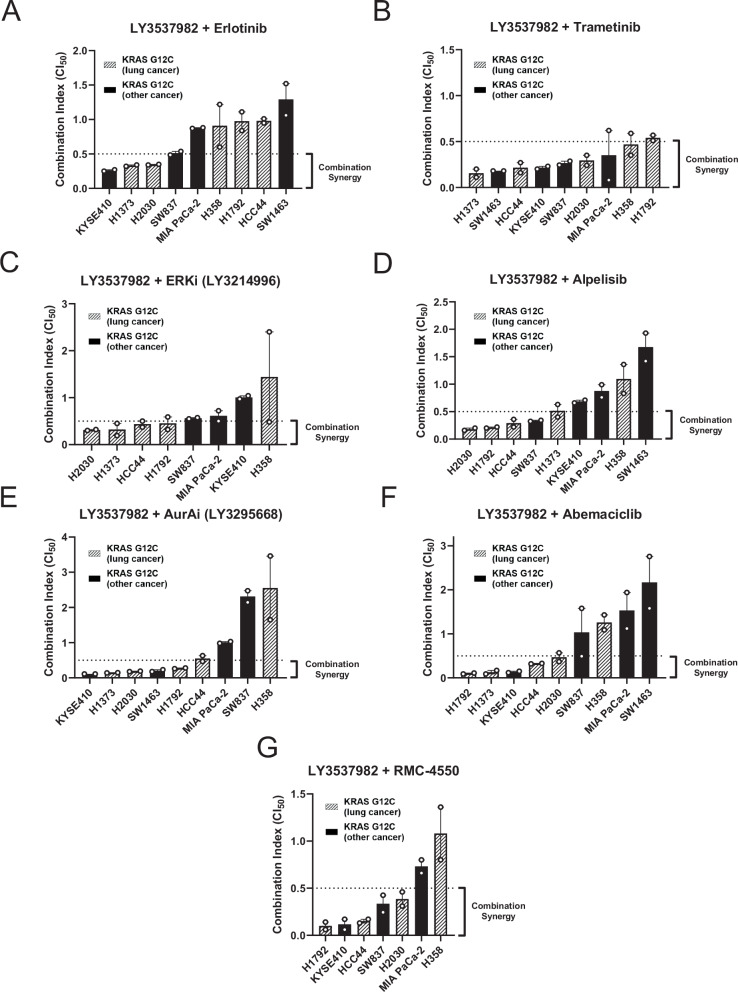
Olomorasib in combination with various therapeutic partners is effective in vitro. A cancer cell line panels was treated with olomorasib in combination with **A** erlotinib (EGFR), **B** trametinib (MEK1/2), **C** ERK inhibitor (LY3214996) **D** alpelisib (PI3K) **E** Aurora A inhibitor (LY3295668) **F** abemaciclib (CDK4/6) and **G** RMC-4550 (SHP2) and subjected to cell proliferation combination analysis (CI_50_). Cell lines are indicated in the Figure. CI ≤ 0.5 (black-dotted line) = synergistic, between 2 and 0.05 = additive, >2 = antagonistic. Source data are available.

Given the frequency of resistance to single-agent treatment and evidence of compensatory mechanisms to overcome RAS inhibition^34^, combination therapies may be needed for patients with KRAS^G12C^-mutant cancers. To systematically screen for potential combination partners, a panel of KRAS^G12C^-mutant cell lines were treated with olomorasib in combination with relevant potential therapeutic partners, including EGFR inhibitor (erlotinib), SHP2 inhibitor (RMC4550), CDK4/6 inhibitor (abemaciclib), ERK1/2 inhibitor (LY3214996), PI3K inhibitor (alpelisib), AurA inhibitor (LY3295668) and MEK inhibitor (trametinib), and the resultant combination index at 50% inhibition (CI_50_) calculated. In most cell lines tested, combination treatments with olomorasib resulted in synergistic (CI_50_ < 0.5) activity (Fig. 2). Supplementary Table 2 further summarizes the CI_50_ for additional therapeutic agents in combination with olomorasib. Selected combinations emerging from this screen were subsequently evaluated in independent cellular and in vivo models

### Olomorasib inhibits KRAS signaling and tumor growth in KRAS^G12C^-mutant xenograft and PDX models

**Fig. 3:**
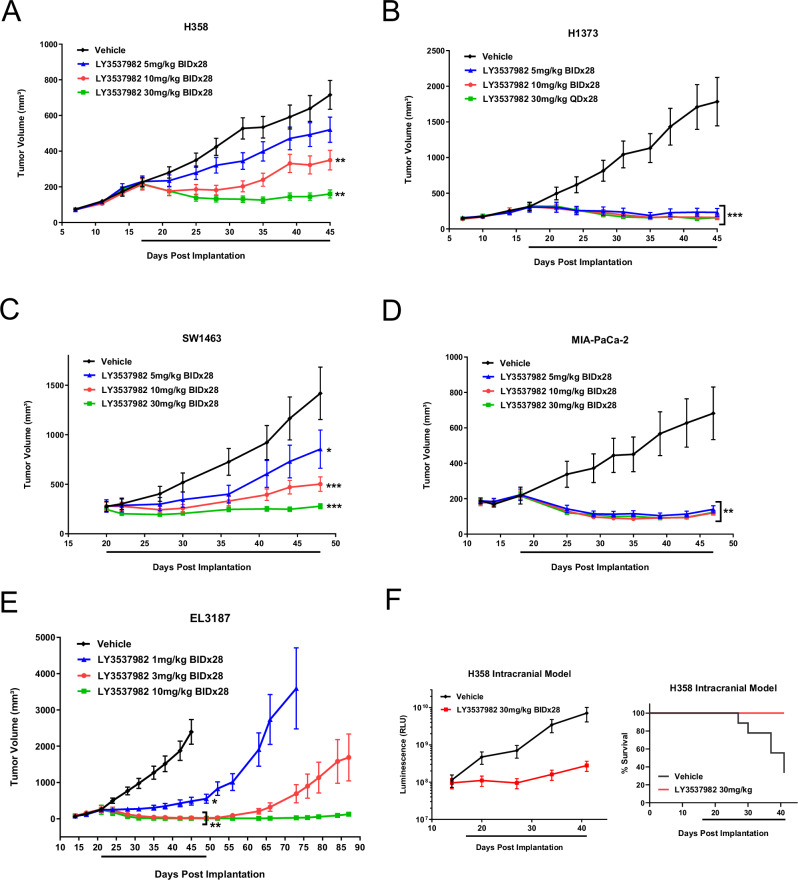
Olomorasib efficacy in *KRAS*^*G12C*^ mutant xenograft, PDX and intracranial cancer models. **A–D** Cancer cell lines (*n* = 6 biological replicates), **E** patient-derived xenograft tumors (*n* = 5 mice) and **F** H358 cells (*n* = 9 mice) were implanted into the flank of immune-deficient mice or intracranially (F). When tumors reached a prespecified burden, mice were randomized to each treatment group and then treated as described in the methods. Tumor volume was measured at the indicated time points. The black line represents the treatment period. Statistical analysis was performed at the end of treatment and is represented in Supplementary Table 3. mpk = mg/kg. *p < 0.001, **p < 0.0001. Data are presented as mean ± SEM. Statistical significance was assessed using a one-tailed ANOVA. Exact *p*-values and sample sizes are indicated in the figure or supplementary tables. Source data are available.

Next, we assessed the effect of olomorasib on KRAS signaling and tumor growth in KRAS^G12C^-mutant xenograft and PDX models. Following tumor formation, mice were treated once with 30 mg/kg olomorasib and tumors analyzed at the indicated time points for RAS activity and pERK levels. Olomorasib significantly (*p* < 0.05) reduced in vivo RAS activity, with peak inhibition of approximately 80% or greater in all three xenograft models (Supplementary Fig. 3A). Similarly, significant pERK inhibition was observed at most time points tested, up to 24 h (Supplementary Fig. 3B). To evaluate the anti-tumor activity of single-agent olomorasib in xenograft models of NSCLC (H358 and H1373), pancreatic (MIA-PaCa-2), and colorectal (SW1463) cancers, mice were dosed twice daily (BID) for 28 days with olomorasib. Subsequent tumor volume and mouse body weight were measured at the indicated time points. Dose-dependent responses with robust tumor growth inhibition and regression were observed in mice treated with 10 mg/kg or 30 mg/kg olomorasib twice daily (Fig. 3A–D, Supplementary Table 3). Additionally, olomorasib was evaluated in an NSCLC PDX model harboring mutant KRAS^G12C^. Consistent with the results from the xenograft models, olomorasib demonstrated potent anti-tumor activity, leading to near-complete tumor regression in the PDX model (Fig. 3E, Supplementary Table 3). We also examined the efficacy of olomorasib in an intracranial tumor model of luciferase tagged-NCI-H358. Following tumor formation, olomorasib was dosed BID for 28 days at 30 mg/kg, and tumor burden was measured at the indicated time points. As with the xenograft models, olomorasib demonstrated a reduction in tumor burden (total flux; Fig. 3F). Overall survival (OS) on Day 40 was 100% in the 30 mg/kg arm compared to 33% in the vehicle arm (Fig. 3F). In all models examined olomorasib was well-tolerated indicated by steady or increased body weight in mice (Supplementary Fig. 2). Taken together, these studies demonstrate robust single agent efficacy of olomorasib in xenograft, PDX and intracranial models, coupled with rapid inhibition of RAS activity and pERK signaling in vivo.

### Olomorasib in combination with other treatments significantly increased efficacy in KRAS^G12C^-mutant xenograft cancer models

**Fig. 4:**
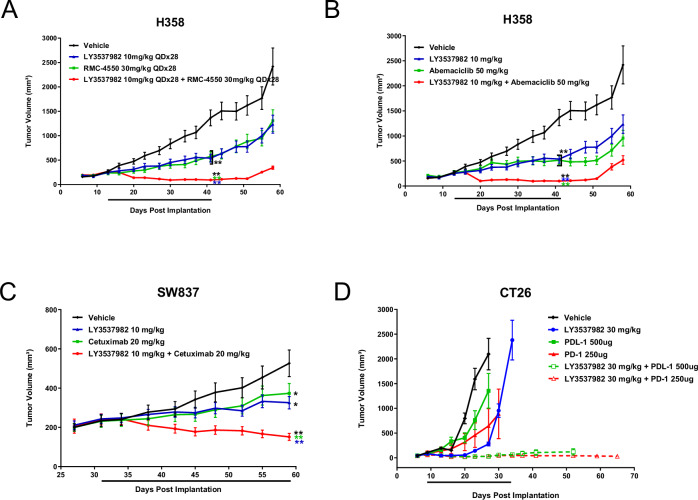
Olomorasib combination efficacy with various therapeutic partners in *KRAS*^*G12C*^ mutant cancer xenograft models. Indicated cell line-derived xenograft (CDX) cancer models were implanted and then treated with **A** olomorasib alone and in combination with RMC-4550 (SHP2 inhibitor) *n* = 4 mice, **B** abemaciclib **C** cetuximab, and **D** PDL-1 and PD-1 inhibitors. (B and C) *n* = 5-6 mice, (D) *n* = 10 mice. Tumor volume was measured at the indicated time points. The black line represents the treatment period. Statistical analysis was performed at the end of treatment and is represented in Supplementary Tables 4 and 5. mpk = mg/kg. *p < 0.001, **p < 0.0001. Data are presented as mean ± SEM. Statistical significance was assessed using a one-tailed ANOVA. Exact *p*-values and sample sizes are indicated in the figure or supplementary tables. Source data are available.

Based on the in vitro combination studies and knowledge of clinically relevant therapeutic approaches, we examined the combinatorial efficacy of olomorasib and abemaciclib, alpelisib, RMC4550, AurA kinase inhibitor, cetuximab, ERK inhibitor, or PD-1 and PD-L1 inhibitor in KRAS^G12C^ mutant lung or colorectal cancer xenograft or syngeneic models. Following tumor formation mice were treated with the indicated dose of olomorasib and the therapeutic partner. Tumor volume and body weight were measured at the indicated time points, as in the single-agent in vivo studies. Compared to vehicle control all single agent treatments significantly (*p* < 0.05) decreased tumor volume in the models examined within the treatment period (Fig. 4, Supplementary Fig. 4**and** Supplementary Tables 4 and 5). Olomorasib in combination with all partners significantly inhibited (*p* < 0.05) tumor growth compared to either single agent or control. Therapeutically additive or synergistic benefits (Bliss independence) were observed in all treatment groups, including olomorasib in combination with PD-1 (additive) and PD-L1 (synergistic) (Fig. 4 and Supplementary Fig. 4). All combinations induced significant tumor regression which, in most models, was sustained even after the treatment was stopped. In the mouse syngeneic CT26 model engineered with KRAS^G12C^, olomorasib in combination with anti-PD-L1 or anti-PD-1 antibody induced complete tumor regression, in four out of nine and six out of ten animals, respectively (Fig. 4D). These results demonstrated that olomorasib in combination with an immune checkpoint inhibitor, PD-L1 or PD-1 antibody, showed superior anti-tumor activity compared to monotherapy. Importantly, olomorasib in combination with additional therapeutic partners was well-tolerated in all treatment groups, indicated by steady or increased body weight throughout the duration of treatment (Supplementary Fig. 5). Additional in vivo models and combination strategies with olomorasib are described in Supplementary Tables 4 and 5.

### Olomorasib efficacy in KRAS^G12C^ cells harboring clinically relevant second site mutations

**Fig. 5:**
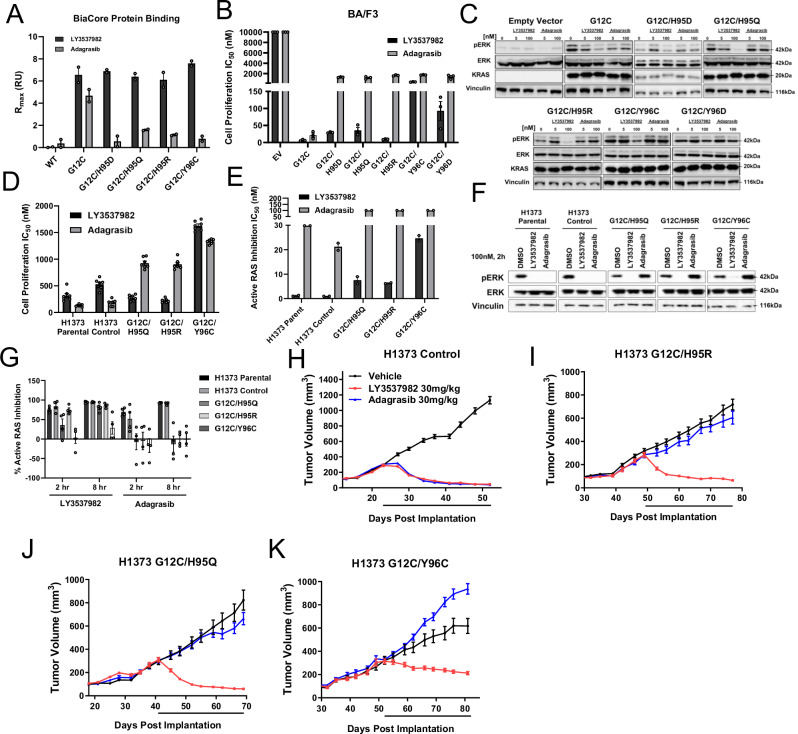
Olomorasib binding and efficacy in models expressing KRAS^G12C^ second site mutations. **A** Olomorasib ability to bind clinically relevant KRAS^G12C^ second site mutations was measured. Ba/F3 cells expressing clinically relevant KRAS^G12C^ second site mutations were treated with olomorasib and **B** effects on cell proliferation and **C** Western blot against indicated proteins was assayed. Cancer cells expressing clinically relevant KRAS^G12C^ second site mutations were treated with olomorasib **D** cell proliferation, **E** RAS inhibition **F** Western blot analysis against indicated proteins **G** %RAS inhibition over time and **H–K** In vivo efficacy of olomorasib in mice harboring the indicated KRAS^G12C^ second site mutation was assayed. Western blots are representative of at least 3 independent experiments. **A**, **E** Data represent mean ± SEM, *n* = 2 biological replicates. **B** *n* = 3 **D** *n* = 7 and **G** *n* = 4 biological replicates. **H** *n* = 6-8 mice, **I** *n* = 6 mice and (**J**, **K**) *n* = 5 mice. Data are presented as mean ± SEM. Statistical significance was assessed using a one-tailed ANOVA. Exact *p*-values and sample sizes are indicated in the figure or supplementary tables. Source data are available.

In addition to bypass mechanisms that confer resistance to KRAS^G12C^ inhibitors^14,21^, several studies have identified second-site KRAS mutations (H95D/Q/R, Y96C/D), following treatment with approved KRAS^G12C^ inhibitors, that disrupt binding of the inhibitor within the switch II pocket region, conferring resistance in the clinic^14,15^. To understand the effect of olomorasib on KRAS within the context of second-site mutations, we examined olomorasib binding to the KRAS protein in the presence and absence of these mutations by surface plasmon resonance (SPR). In KRAS wild type, both olomorasib and adagrasib showed an R_max_ (maximal relative binding) <1, indicating poor binding of both inhibitors to wild type KRAS, as expected. Comparatively, both olomorasib and adagrasib exhibited high binding affinity to KRAS^G12C^, with R_max_ values of 7.25 and 4.12, respectively. Following introduction of the second site mutations, R_max_ values for olomorasib were 7.02 (H95D), 6.78 (H95Q), 6.10 (H95R), and 7.81 (Y96C), compared to R_max_ values of 1.04, 1.07, 1.65, and 1.03, respectively, for adagrasib (Fig. 5A**and** Supplementary Fig. 6A). These results, consistent with our crystal structure of olomorasib-KRAS^G12C^ showing no strong interactions of olomorasib with H95 or Y96, demonstrate that olomorasib continues to bind mutant KRAS^G12C^ protein tightly in the presence of clinically relevant second site mutations.

We next examined the efficacy and inhibitory activity of olomorasib within the context of clinically relevant second-site KRAS mutations in cells. Ba/F3 cells were transfected with viral vectors expressing mutant KRAS^G12C^ and relevant second-site mutations. Stably expressing cell lines were treated with olomorasib or adagrasib for 96 h and the effects on cell viability, as well as pERK levels, were evaluated. Neither compound had substantial effects on cell viability in the control empty vector cells (Fig. 5B). In contrast, in cells expressing KRAS^G12C^, both olomorasib and adagrasib displayed potent dose-dependent inhibition of cell viability, with IC_50_ values of 6 nM and 20 nM, respectively. By western blot analysis, the inhibitory effect of both compounds on pERK levels was consistent with their effects on cell viability (Fig. 5C). Moreover, in cells expressing H95D/Q/R second site mutations, olomorasib robustly decreased cell proliferation and pERK levels, which were not observed in cells treated with adagrasib (Fig. 5B, C). In cells with Y96C/D second-site mutations, olomorasib also decreased cell proliferation and pERK levels in a mutation-dependent manner, but to a lesser extent than the H95 mutations. As with the H95 mutations, adagrasib treatment did not affect cell proliferation and pERK levels in cells harboring Y96C/D second-site mutations. To further evaluate the effect of olomorasib on KRAS signaling, MEF cells lacking RAS expression (Rasless), were also transfected with vectors harboring either the single site KRAS^G12C^ mutation, or second-site KRAS mutations (with G12C background) and treated as before with olomorasib, adagrasib or sotorasib. KRAS protein expression was confirmed by western blot, and pERK activity was measured. In parental (RASless) cells, neither olomorasib, adagrasib, nor sotorasib showed activity. As in the prior Ba/F3 model, transfection with either single-site KRAS^G12C^ or KRAS^G12C^ plus second-site mutations revealed differential sensitivity to KRAS inhibitors. Cells treated with olomorasib demonstrated greater sensitivity, as measured by pERK inhibition, compared with adagrasib. In contrast, cells harboring Y96 second-site mutations exhibited a reduced response to sotorasib, both in vitro (pERK inhibition; Supplementary Fig. 7D) and in vivo (Supplementary Fig. 8), consistent with previous reports^35^. Notably, relative to adagrasib and sotorasib, olomorasib maintained greater activity across all tested second-site mutations, indicating a broader inhibitory profile (Supplementary Figs. 7 and 8).

Next, we examined the activity of olomorasib in NCI-H1373, a model of KRAS^G12C^ NSCLC with CRISPR knock-in second-site mutations, both in vitro and in vivo. Consistent with our previous results, we observed retained antiproliferative activity, as well as inhibition of active RAS and pERK levels following treatment with olomorasib relative to CRISPR-matched control, not seen in adagrasib-treated cells harboring second site mutations (Fig. 5D-G, Supplementary Fig. 6B). In xenograft studies, both olomorasib and adagrasib significantly (*p* < 0.05) decreased tumor volumes in NCI-H1373 xenograft models (Fig. 5H), while only olomorasib reduced (*p* < 0.05) tumor volumes in mice harboring second site mutations, resulting in tumor regression in two of the xenografts (Fig. 5I-K). Tumor regression was not observed in adagrasib-treated mice with KRAS second-site mutations. In all models examined, treatment was well-tolerated, indicated by steady or increased body weight in the mice (Supplementary Fig. 9A-D) and drug exposure is summarized in Supplementary Fig. 9E.

### Molecular characteristics of KRAS^G12C^-mediated drug resistance

**Fig. 6:**
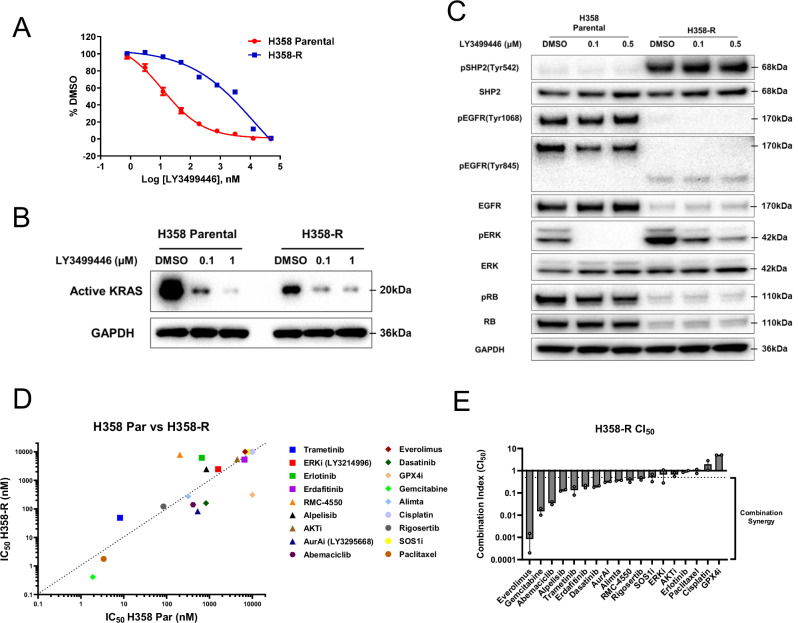
Resistance to KRAS^G12C^ inhibitors induces targetable acquired dependence in cancer cells. **A** H358 cells were treated with LY3499446 an analog of olomorasib (**NCT04165031)** until resistance was observed. Cross-resistance to olomorasib was confirmed (*n* = 3 biological replicates). Western blot against **B** active KRAS and **C** additional indicated proteins was performed Western blots are representative of at least 3 independent experiments. LY3499446-resistant cells were treated with a panel of anti-cancer drugs and **D** IC_50_ (data presented is average of replicate data) and **E** CI_50_ were measured. (*n* = 2 biological replicate, replicate data including SEM are available in the Source Data). All Source data are available.

We next investigated the molecular effects of drug-mediated resistance in vitro and therapeutic strategies to overcome resistance to single-agent KRAS^G12C^ inhibitors. H358 and MiaPaca-2 cells were treated with an analog of olomorasib (**NCT04165031**^2^) until resistance was observed. Resistance to olomorasib in these models was confirmed (Fig. 6A**and** Supplementary Fig. 10A). In resistant H358 cells, we observed reduced levels of active KRAS, increased pSHP2 (Y542), and decreased pEGFR (and total EGFR) (Fig. 6B and C). Compensatory SHP2 activation has been shown to reactivate RAS pathway signaling following RAS inhibition^36^. In KRAS^G12C^ inhibitor-resistant MiaPaca-2 cells we observed increased pEGFR (Y1068) and pERK (untreated lanes) (Supplementary Fig. 10B), suggesting diverse resistance mechanisms. Following onset of resistance to various KRAS^G12C^ inhibitors, we performed a compound screen in KRAS^G12C^ inhibitor-resistant and sensitive cell lines, to identify therapeutic vulnerabilities and resistance pathways. Screened compounds were selected based on the mechanism of action. Interestingly, both H358 and MiaPaca-2 KRAS^G12C^ inhibitor-resistant cells maintained sensitivity to chemotherapies gemcitabine and paclitaxel. Additionally, compounds like dasatinib and AurA inhibitor demonstrated greater potency in both H358 and MiaPaca-2 resistant cells, compared to parental cells (Fig. 6D, Supplementary Fig. 10C). Combination with several of these inhibitors, including gemcitabine, dasatinib, and inhibitors of the PI3K pathway also resensitized the olomorasib-resistant cells to olomorasib treatment (Fig. 6E, Supplementary Fig. 10D). These results highlight potential therapeutic vulnerabilities to overcome resistance to KRAS^G12C^ inhibitors.

## Discussion

The discovery of drugs capable of targeting KRAS^G12C^ has shifted the treatment landscape for many non-small cell lung cancer (NSCLC) patients harboring these mutations, as well as in other KRAS^G12C^-driven cancers^1,2,7,9^. Despite these advances, few effective treatment options remain available, and therefore, improved targeting of these proteins remains an important goal for therapeutic development. Two major limitations present in currently approved KRAS^G12C^ inhibitors are 1) high-grade liver toxicity and 2) incomplete target occupancy^10,11^. Olomorasib is an oral, highly selective, and potent, covalent inhibitor of KRAS^G12C^. Early clinical trial results of olomorasib have demonstrated favorable safety profiles, including the absence of high-grade liver toxicity^37^. Moreover, olomorasib was effective and well-tolerated in patients who had previously received KRAS^G12C^ inhibitors^37^. Structurally, the 8-membered heterocycle of olomorasib constrains the molecule’s geometry to efficiently deliver the acrylate to C12. Conformations of the bound C12 are in complete agreement with the most favorable, low-energy unbound C12 conformations, indicating no strain after formation of the covalent bond. The high-affinity interaction of olomorasib with KRAS^G12C^ has a target occupancy of >90% during the 24 h dosing period (compared to less than 80% for adagrasib^10,29^, and 88% for sotorasib^30,38^) with robust dose-dependent inhibition of RAS activity, RAS-GTP, and pERK expression. RAS-mediated gene expression was similarly decreased in KRAS^G12C^-mutant lung and pancreatic cancer models. Moreover, we observed olomorasib monotherapy robustly decreased cell proliferation in KRAS^G12C^ cell models.

In xenograft and PDX models, as in our in vitro models, olomorasib monotherapy decreased tumor growth, and both RAS activity and pERK level in models of KRAS^G12C^-mutant cancers. Olomorasib-mediated tumor growth inhibition was both dose and schedule-dependent (twice daily vs once daily (BID v. QD)). In mice BID we observed enhanced tumor growth inhibition compared to QD treatment. Importantly, the change in dosing schedule did not affect tolerability in mice, indicated by steady body weight regardless of dose and treatment schedule. Maximal inhibition of RAS activity and pERK were observed at the 30 mg/kg dose, and within eight h of treatment. In an intracranial xenograft model, we also observed single-agent efficacy and improved overall survival of mice, suggesting potential CNS penetration and efficacy of olomorasib in brain metastatic KRAS^G12C^-mutant lung cancers.

Previous studies of KRAS^G12C^ inhibition have demonstrated increased protein expression of key RAS and MAPK pathway regulators following KRAS^G12C^ inhibition, hypothesized to compensate for RAS inhibition^14^, providing a rationale for frontline combination therapies with KRAS inhibitors. Moreover, the biological necessity of reactivating RAS downstream signaling further underscores the need for upfront combination strategies in this setting. To this point, we observed additive or synergistic benefit when adding mechanism-based inhibitors to olomorasib treatment in vitro and in vivo. A combination of cetuximab (EGFR inhibitor) plus olomorasib showed synergism in an in vitro model of KRAS^G12C^ colorectal cancer and resulted in tumor regression and delayed tumor growth in vivo. A combination of olomorasib (LY3537982) plus cetuximab is currently under clinical investigation (**NCT04956640**).

Current SOC treatment in NSCLC targets the immune checkpoint mediators PD-L1 and PD-1^39^. However, due to liver toxicity associated with currently approved KRAS^G12C^ inhibitors, combination therapies including PD-L1/PD-1 inhibitors in this setting are challenging^11^. In contrast to adagrasib and sotorasib, monotherapy olomorasib has not demonstrated high-grade liver toxicity. Consistent with this, olomorasib potently inhibited tumor growth in combination with either PD-1 or PD-L1 inhibitors. These combinations were well-tolerated as indicated by steady body weight in mice. Combination of olomorasib with pembrolizumab with and without chemotherapy is currently under clinical investigation (NCT04956640 and NCT06119581)^26^. These findings may support a prioritized framework for combination development, with cetuximab + olomorasib in colorectal cancer and pembrolizumab + olomorasib in NSCLC as clinically actionable strategies.

Frequent resistance mechanisms to KRAS^G12C^ inhibitors include, in addition to KRAS amplification^14^, second-site KRAS mutations^14,35^. Second site mutations at H95 (H95D/Q/R) have been shown to mediate resistance to adagrasib^35^, as well as mutations at Y96 (Y96C/D) through disruption of inhibitor binding^35^. We observed in olomorasib binding assays, regardless of mutation present (with KRAS^G12C^ mutation present) that olomorasib binds KRAS^G12C^ tightly. In contrast, adagrasib showed tight binding to KRAS^G12C^ single mutation, and substantially decreased binding to all double mutants examined. Consistent with these observations and given that olomorasib does not interact directly with either H95 (as observed for adagrasib) or Y96, it potently decreased cell viability and pERK expression in all KRAS^G12C^ double mutants (H95D/Q/R and Y96C/D) expressed in Ba/F3 cells, as well as in RASless MEF cells following CRISPR knock-in of the double mutations. In vivo, olomorasib robustly decreased tumor progression in all second-site mutation models examined.

In summary, our results describe the preclinical efficacy and safety profile of olomorasib, a novel highly potent and selective KRAS^G12C^ inhibitor with >90% target occupancy and potential CNS penetration. Olomorasib alone and in combination with additional therapeutic partners decreased tumor growth in xenograft and PDX models of KRAS^G12C^-driven cancers, as well as in tumor models with clinically relevant second-site KRAS^G12C^ mutations. Olomorasib is currently under clinical investigation as a monotherapy and in combination with additional mechanism-based therapeutic agents (**NCT04956640**), as well as with first-line SOC pembrolizumab in the SUNRAY-01 global phase 3 clinical trial (**NCT06119581**)^26^.

## Methods

All animal studies were conducted in accordance with the American Association for Laboratory Animal Care institutional guidelines. All in vivo experimental protocols were approved by the Eli Lilly and Company animal care and use committee.

Specific NMR assignments were determined using HSQC experiments.

### In vitro cell proliferation and combination treatment

All tested cancer cell lines were obtained from ATCC (Manassas, VA), unless indicated otherwise (H358: CRL-5807; CT26: CRL-2638; SW837: CCL-235; SW1463: CCL-234; MIA PACA-2: CRM-CRL-1420; H1373: CRL-5866; H1792: CRL-5895; H2030: CRL-5914; H2122: CRL-5985; SW1573: CRL-2170), HCC44 and KYSE-410 (DMZ – KYSE-410: ACC 381; HCC-44: ACC-534), with the following exceptions: LXFA-983L (obtained from Oncotest, Charles Rivers Laboratories, Wilmington, MA), EL3187 (developed at Lilly from a patient derived xenograft model). LU99 (purchased from Japanese Collection of Research Bioresources Cell Bank; JCRB0080). Resistant cell lines were generated by culturing parental cells in medium containing the indicated KRAS^G12C^ inhibitors. The concentration of KRAS^G12C^ inhibitors was gradually increased to the desired concentration and resistance was confirmed by CTG assay. Resistant cells were kept in the medium without the compound for 24 h prior to the single or combination treatment. All cell lines have a KRAS^G12C^ mutation, except for A549 which harbor a KRAS^G12S^ mutation. All commercially available cell lines were maintained in their respective recommended medium from the supplier. The EL3187 cell line was grown in DMEM + 10% FBS. For in vitro 2D cell proliferation assays, 2000 cells/well were plated in 96-well cell culture plates (Corning/#3603 black/clear flat bottom) in 80 µL/well of growth media. Cells were incubated overnight in a 37 °C, 5% CO_2_ incubator with humidity for all cell lines except for SW756 (CRL-3584), SW837 (CCL-235), and SW1573 (CRL-2170), which require no CO_2_. The following day, cells were treated with compounds, either a single or a combination treatment. First, the testing compounds were serially diluted in DMSO, then diluted into media to 5X with 1% DMSO, and finally added to cells in media to dilute to 1X. Cells were incubated at 37 °C for the indicated time. The highest concentration of most compounds was 10 µM with a total of 10 concentration points by 1:3 serial dilution, and the final DMSO concentration was 0.4%. CTG was performed according to the manufacturer’s protocol (Promega #G75723).

Absolute IC_50_s generated by a 4-parameter logistics model for the single and combination treatments, followed by combination indexes for each combination treatment and cell line. The combined IC_50_s were adjusted based on the total concentration of each compound when added together. The biological interpretation of the combination index is as follows: synergistic if the combination index <0.5, additive if the combination index is between 0.5 and 2, and antagonistic if the combination index is >2.

DU315-6 MEF RASless cell line was provided by Mariano Barbacid^33^.

### Proteome-wide cysteine profiling sample preparation

The mass spectrometry-based proteome-wide cysteine selectivity profiling was performed as described previously^31^. Two biological replicates of H358 (CRL-5807) cells (approximately 90% confluence in T-225 cell culture flask) were treated with olomorasib the indicated concentrations or dimethyl sulfoxide (DMSO) at 37 °C for 4 h. After incubation, the cells were harvested and lysed with 500 µL lysis buffer (DPBS, 1% NP-40) on ice for 10 min. The lysate was centrifuged at 20,000 x *g* for 10 min and the supernatant was collected. The protein concentration was determined using the BCA protein assay (Pierce Cat # 23225) and diluted to 8 mg/mL with lysis buffer. Next, 500 µL cell lysate was mixed with 500 µL 12 M urea in 200 mM Tris, pH 8, then labeled with 100 µM thiol probes (Light probe LSN3399592 for DMSO-treated samples and heavy probe LSN3399593 for olomorasib-treated samples) at room temperature in the dark for 1 h. The reaction was quenched with 100 mM DTT at 65 °C for 20 min. 1 mL of probe LSN3399592-labeled samples and 1mL of probe LSN3399593-labeled samples were combined, then aliquoted into two portions (1mL each). Each aliquot was loaded onto a pre-equilibrated Econo-Pac 10DG column (equilibrated with gel filtration buffer: 20 mL 2 M urea in 50 mM Tris, pH 8). The columns were washed with 1.5 mL gel filtration buffer and then eluted with 1.5 mL gel filtration buffer. The collected eluates from the same sample were combined and digested with 40 µL of 0.5 µg/µL sequencing-grade modified trypsin (Promega Cat # V5111) at 37 °C overnight. The digested sample was diluted with 340 µL of 10X binding buffer (10X PBS, 10% Triton, 5% Tergitol, 10 mM EDTA) for streptavidin enrichment.

Each biological replicate was divided into two equal aliquots of 700 μL. Each aliquot was loaded onto Agilent Technologies Streptavidin Tips (Cat # G5496-60010) that were compatible with Agilent Bravo AssayMap using the vendor-defined application: “Affinity Purification 1.0”. Briefly, the tips were washed 2X with PBS, first with 100 μL at 300 μL/min, and second with 50 μL at 10 μL/min. The digested samples were then loaded onto the tips at 10 μL/min. The tips were washed twice with double-distilled water (250 μL) at 10 μL/min and subsequently eluted with 1/1 (v/v) acetonitrile/0.1% formic acid (50 μL) at 10 μL/min. The resulting 20 samples were then dried to complete dryness using a speed-vacuum system.

### Proteome-wide cysteine profiling LC-MS/MS and data analysis

Nano-LC-MS/MS analysis was performed on a Q Exactive HF Mass Spectrometer (Thermo Fisher Scientific, Hanna-Bremen, Germany), coupled to a Dionex UltiMate 3000 (Germering, Germany) RSLC nano system. Two mobile phase solvent systems were utilized for the liquid chromatography: 1) Mobile Phase A (formic acid or FA/H_2_O 0.1/99.9, v/v) and 2) Mobile Phase B (FA/acetonitrile or ACN 0.1/99.9, v/v). Samples were reconstituted in 50 μL 0.1% formic acid aqueous solution and analyzed two times using nano-LC-MS/MS (10 μL/injection). A total of 4 nano-LC-MS/MS analyses were performed for each biological replicate. Each 10 μL injection was loaded onto an Acclaim PepMap® 100 trap column with nanoViper connections (inner diameter of 100 μm and length of 2 cm with C18 particles of 5 μm carrying 100 Å pore sizes) from Dionex/Thermo Fisher Scientific at 7 μL/min for six min using Phase-A. For the liquid chromatographic separation of the concentrated peptides, the trap column was then switched to align with the analytical column, an Acclaim® PepMap RSLC with nanoViper connections (inner diameter of 75 μm and length of 25cm, with C18 particles of 2 μm and 100 Å pore size). The loaded peptides were eluted at 250 nL/min using a varying mobile phase gradient, from 99/1 A/B (v/v) to 75/25 A/B (v/v) for 124 min, next from 75/25 A/B (v/v) to 65/35 A/B (v/v) for 36 min, then from 65/35 A/B (v/v) to 20/80 A/B (v/v) for 4 min, and finally keeping the same mobile phase composition for the next 10 min. Nano-LC mobile phase was introduced into the mass spectrometer using an EASY-Spray source. The nanospray was operated by applying an ion spray voltage of 2.49 kV onto the spray tip while keeping the ion transfer capillary at 300 °C. The mass spectrometer method was operated under data dependent (“Full MS / dd-MS2 – TopN”) mode, programmed to select top 12 most intense ions in a full MS scan with vendor defined parameters (Microscans 1, Resolution 60k), AGC target 1E6, Maximum IT 100 ms, Number of scan ranges 1, Scan range 300 to 1700 m/z, and Spectrum data type “profile”, and then to perform data-dependent MS/MS scans with vendor defined parameters (Microscans 1, Resolution 15k), AGC target 5E5, Maximum IT 80 ms, Loop count 12, MSX count 1, Isolation window 2.0 m/z, Fixed first mass 100 m/z, NCE 25.0, Stepped NCE 24, 30, 36, and Spectrum data type “centroid”. The respective data-dependent settings were set with vendor defined parameters: Intensity threshold of 3.8 ×105, Apex trigger as “-”, Charge exclusion as “-”, Peptide match as “preferred”, Exclude isotopes as “on”, Dynamic exclusion of 40.0 s, and If idle “do not pick others”. Data were recorded using Thermo Xcalibur (4.0.27.19) software (Copyright 1998 to 2015, Thermo Fisher Scientific Inc.).

To identify and quantify “light” and “heavy” cysteine (Cys)-labeled peptides and then to determine the “heavy/light” ratios for each peptide identified, all resulting files were analyzed using the Thermo Proteome Discoverer Software suite 2.4.0.305. The MS/MS spectra were searched with SEQUEST HT against an in silico tryptic digest of homo sapiens (human) proteins database (FASTA format) from the UniProt sequence database (version June 2016), which was modified in-house to carry KRASG12C. To identify cysteine residues modified with thiol-reactive isotopic probes, potential modifications of +554.343 Da, representing “light” modification (C26H46N6O7) and of +560.357 Da, representing “heavy” modification (C2113C5H46N615NO7) were incorporated into the SEQUEST HT configuration. Peptide identifications were filtered with Percolator to yield a 1% FDR. The resulting set of peptides “PeptideGroups.txt” file was then imported to JMP® 14.1.0 (64-bit, Microsoft Windows 7 Enterprise 64-bit, Service Pack 1, Copyright©2015 SAS Institute Inc.) for additional processing. Normalized “H/L” ratios (i.e., “heavy intensity”/“light intensity”) of each identified peptide were used to calculate log2 [H/L] ratios.

### Target occupancy sample preparation

SW1463 cells (5 million) were first pre-treated with olomorasib at the indicated concentrations or DMSO at 37 °C. After treatment, cells were harvested at the indicated time points and lysed with DPBS, 1% NP-40. Cell lysates were collected by centrifugation and quantified by the BCA Protein Assay Kit (Pierce, Cat# 23225). Cell lysates were diluted to 5 mg/mL with 6 M Urea, 100 mM Tris, pH 8. 500 µg of lysates were incubated with 10 mM Dithiothreitol (DTT) at 65 °C for 15 min and followed by 50 mM Iodoacetamide at 37 °C for 30 min. After incubation, the buffer was changed to 50 mM Tris, pH 8, with a Zeba Spin Desalting Plate (Thermo Scientific, cat# 89807). Lysates were digested with 5 µg Trypsin/Lys-C Mix (Promega, cat# V5071) at 37 °C overnight. The digested samples were immunoprecipitated with the biotinylated anti-RAS peptide (GGVGK) antibody (Lilly, ELI280-1D1) using the Agilent Bravo AssayMap system. Briefly, Streptavidin tips (Agilent, cat# G5496-60010) were first washed with 100 µL PBS at 100 µL/min and then with 50 µL PBS at 10 µL/min. 10 µg antibody in 100 µL PBS was loaded onto each tip at 3 µL/min. The tips were washed with 50 µL PBS at 10 µL/min. The digested samples were mixed with isotope-labeled internal standard peptides (5 nM LVVVGAC(cam)GVGK(13C615N2) (CPC Scientific, Sunnyvale, CA, ID925863) and loaded onto the tips at 2 µL/min. The bound peptides were washed with 50 µL PBS at 10 µL/min and then eluted with 50 µL 0.1% TFA at 2.5 µL/min.

### Target occupancy LC-MS/MS and data analysis

In order to calculate the KRAS^G12C^ target occupancy for LY3537982, the concentration of KRAS^G12C^ tryptic peptide, LVVVGAC(cam)GVGK, was measured with a targeted LC MS/MS assay. From the measured peptide concentrations, a percentage target occupancy value was calculated by normalization of DMSO-treated samples using the following formula:

$${{\rm{TO}}}=100{{\rm{x}}}\left(1\!-\!({[{{\rm{G}}}12{{\rm{C}}}\!:\!{{\rm{cam}}}]}_{{{\rm{LY}}}}/({[{{\rm{G}}}12{{\rm{C}}}\!:\!{{\rm{cam}}}]}_{{{\rm{DMSO}}}}))\right.$$

HPLC-MS analysis was performed with a Vanquish HPLC system (Thermo Fisher Scientific, San Jose, CA) interfaced to a Q-Exactive mass spectrometer (Thermo Fisher Scientific, Bremen, Germany) operating in targeted MS/MS mode. Mobile phase A was composed of water containing 0.1% formic acid. Mobile phase B was comprised of acetonitrile containing 0.1% formic acid. At the starting mobile phase composition of 1% B, 40 μL of each sample was injected onto a C18 HPLC column (Thermo Acclaim RSLC 120 C18 2.2 µm 120 A 2.1 ×150 PN: 071399) at a flow rate of 0.6 mL/min. Following a 1 minute ramp to 5% B, the gradient was increased to 50% over four minutes. Operating in a targeted “PRM” mode, the mass spectrometer targeted the KRAS^G12C^ peptides LVVVGAC(cam)GVGK, and its 13C615N2 isotope-labeled internal standard for fragmentation and high-resolution product ion analysis (precursor m/z values: 496.56, and 499.23). MS data analysis was performed with Skyline (64-bit) 4.1.0.18169 (MaCoss Lab. Department of Genome Sciences, University of Washington) and the percentage target occupancy was calculated with Microsoft Excel.

### pERK assay

The cells were plated in 90 µl of appropriate growth medium at 20,000-40,000 cells/well in a 96-well tissue culture plate (Falcon, Cat. No. 353377) and incubated overnight at 37 °C in 5% CO_2_. The day after plating, the cells were treated with 10 µl of serially diluted olomorasib (in Opti-MEM plus 10% DMSO) and incubated at 37 °C for the indicated time. pERK activity was measured using AlphaLISA SureFire Ultra kit (Perkin Elmer, MPSU-PTERK-K500) and according to the manufacturer’s protocol.

### Active RAS ELISA assay

The cells were plated in 90 µl of appropriate growth medium at 20,000-40,000 cells/well in a 96-well tissue culture plate (Falcon, Cat. No. 353377) and incubated overnight at 37 °C in 5% CO_2_. The day after plating, the cells were treated with 10 µl of serially diluted olomorasib and incubated at 37 °C for the indicated time. Following incubation, the ELISAs were performed using the Active Motif Ras GTPase Chemi Elisa Kit (Cat. No. 52097, Lot No. 21620014) according to the manufacturer’s protocol

### In vitro kinetic assay

The KRASG12C (amino acids 1-188) was expressed in-house using E. coli as an N-terminal 6x-histidine (HIS) fusion followed by a TEV cleavage site. Purification included the “loading” of the protein with the guanosine diphosphate (GDP) nucleotide by overnight dialysis in a buffer containing excess GDP nucleotide. Loading was confirmed by liquid chromatography-mass spectrometry (LCMS). The enzyme was stored at −80 °C until the assay. Reactions were initiated by adding the compound to the protein and subsequently quenched by adding 1 reaction volume of 0.4% formic acid (0.2% final). Protein modification was monitored by Agilent 6546 QTOF operating in positive ion mode. After the entire time course was completed, the assay plate was sealed and then loaded onto the LCMS for data collection. For each time point and compound concentration, four technical replicates were used. Data analysis was performed using the Masshunter V7 software (Agilent).

### Surface plasma resonance

Biotinylated KRAS^G12C^ proteins (produced in-house) were immobilized at equal levels (~600 response units, RU) on a streptavidin (SA) sensor chip (Cytiva, #BR100531). Equal loading was confirmed across all flow cells to ensure consistent surface density for comparative analysis.

The assay was performed using a BIACORE T200 instrument. KRAS^G12C^ inhibitors were diluted in running buffer (HBS-P + ; Cytiva, #BR100671) and injected at five concentrations in a 3-fold serial dilution series: 0.0111 µM, 0.0333 µM, 0.1 µM, 0.3 µM, and 0.9 µM. Binding responses were recorded in resonance units (RU), which are proportional to the mass of analyte bound to the immobilized protein.

Theoretical R_max_ values were calculated based on the analyte's molecular weight and the protein's immobilization level. Observed R_max_ values obtained from curve fitting using a 1:1 Langmuir binding model were consistent with theoretical predictions, confirming the validity of the binding interactions.

### Protein expression and purification

The gene for human KRAS (RefSeq NP_004976). The clone of cysteine light (G12C/C51S/C80L/C118S), corresponding to amino acids 1–169, was TOPO-cloned into a custom pET26b Escherichia coli expression vector (Novagen) in-frame with a N-terminal hexahistidine affinity tag. Bacterial BL21(DE3) was used as the expression host, and the induction of protein expression was performed in 2X TY medium with 1 mM isopropyl β-D-1-thiogalactopyranoside at 37 °C for 5 h. Cells were collected and stored at –80 °C for subsequent protein purification. The frozen cell pellets were lysed and purified using affinity, Mono Q ion-exchange, and incubated with 3 M excess compound LY3537982 at 4 °C overnight. The final step was purified using size-exclusion chromatography, with the His-tag removed for crystallization.

### Protein crystallization

15.5 mg/ml Concentrated Protein in 200 mM HEPES pH7.5, 150 mM NaCl, 1 mM DTT was crystallized using the sitting-drop vapor-diffusion method in a 96-well tray at 21 °C. Crystals were obtained by mixing 0.5 μl of protein with 0.5 µl reservoir solution (0.1 M Tris HCl 7.5, 26.7%PEG 4 K, 200 mM Calcium Chloride). After 10 days, the rectangle-shaped crystals were harvested into cryoprotectant (20% Ethylene glycerol and reservoir) and flash-frozen in liquid nitrogen.

### X-ray data collection and structure determination

Diffraction data were collected at the Lilly Research Laboratories Collaborative Access Team (LRL-CAT) beamline at Sector 31 of the Advanced Photon Source. Crystals stored in liquid nitrogen were mounted on a goniometer equipped with an Oxford Cryosystems Cryostream maintained at 100 K. The wavelength used was 0.9793 Å. The diffraction data were indexed and integrated using MOSFLM 7.0.5 and merged and scaled with Scala 3.3 and Truncate 6.5 from the CCP4 6.5 suite. The structure was solved by molecular replacement with PHASER, using a search model derived from the KRAS-G12C GDP structure (4L8G^7^). The initial structure coordinates for the dataset were further refined using REFMAC v.5.8 (CCP4), applying anisotropic temperature factors. Model building was performed with Coot (CCP4) and final structure validation with MolProbity and CCP4 validation tools. Additional data collection and refinement details can be found in Supplementary Table 1.

Protein coordinates and structure factors have been deposited with the Protein Data Bank (https://www.wwpdb.org/) under the access code: 9N44.

### Western blot

For Western blot analysis, 1 × 10⁶ or 8 × 10⁵ cells were seeded in 60 mm tissue culture dishes containing 4 mL of media. The following day, cells were treated with 40 µL of compound diluted in Opti-MEM supplemented with 10% DMSO and incubated at 37 °C for the indicated times. After treatment, cells were washed once with cold PBS and lysed in 1× cold lysis buffer (Cell Signaling Technology, Cat. No. 9803) supplemented with protease and phosphatase inhibitors (Thermo Fisher, Cat. No. 1861281). Lysates were clarified by centrifugation at 15,000 rpm for 15 min at 4 °C, and protein concentrations were determined using the DC Protein Assay Kit (Bio-Rad, Cat. No. 5000112).

Equal amounts of denatured protein were resolved on Tris-glycine gels (Invitrogen, Cat. Nos. XP04205BOX and WXP42026BOX) and transferred to 0.2 µm nitrocellulose membranes using the Bio-Rad Trans-Blot Turbo Transfer System (Cat. No. 1704159). Membranes were blocked in 5% non-fat milk (Bio-Rad, Cat. No. 1706404XTU) in TBS-T (Teknova, Cat. No. T9515) for 1 h at room temperature, followed by overnight incubation at 4 °C with primary antibodies diluted in 3% BSA (Fisher, Cat. No. BP9706100) in TBS-T. After three washes with TBS-T, membranes were incubated with HRP-conjugated secondary antibodies (anti-rabbit IgG, CST Cat. No. 7074; or anti-mouse IgG, CST Cat. No. 7076) diluted in 5% milk/TBS-T for 1 h. Signal was detected using SuperSignal West Pico PLUS chemiluminescent substrate (Pierce, Cat. No. 34577) and imaged on a Fujifilm LAS-4000 system.

### Ba/F3: transfection, cell viability assay, and western blot

KRAS G12C double mutant expression vectors were generated in-house. For transfection, 1 × 10⁶ Ba/F3 cells were electroporated with 10 µg of plasmid DNA using the Neon Transfection System (Invitrogen) at 1600 V, 10 ms, 3 pulses. Cells were transferred to RPMI 1640 medium (Gibco) supplemented with 10% heat-inactivated FBS (Gibco) and 10 ng/mL mouse IL-3 (R&D Systems) and incubated at 37 °C for 48 h. Cells were then selected in 1 µg/mL puromycin (Gibco) until stable growth was observed, followed by adaptation to IL-3–free conditions.

For viability assays, cells were treated with a 9-point, 3-fold serial dilution of KRAS G12C inhibitors starting at 10 µM. Cell viability was assessed using CellTiter-Glo (Promega), and IC₅₀ values were calculated using GraphPad Prism. Each experiment was performed in triplicate.

For Western blotting, 2 × 10⁶ Ba/F3 cells expressing KRAS G12C double mutants were treated with DMSO or KRAS G12C inhibitor for 6 h at 37 °C. Cells were harvested, washed with PBS (Gibco), and lysed in 70 µL of 1% SDS containing 2× HALT protease/phosphatase inhibitor (Thermo Fisher). Lysates were heated at 95 °C for 5 min, sonicated for 10 seconds, and quantified using the Pierce BCA Protein Assay Kit (Thermo Fisher). Equal amounts (20 µg) of protein were resolved on 4–20% Criterion Tris-HCl gels (Bio-Rad) and transferred to nitrocellulose membranes using the Bio-Rad TurboBlot system (high molecular weight setting, 10 min). Membranes were blocked in 5% milk/TBS-T for 1 h and incubated overnight at 4 °C with primary antibodies (Cell Signaling Technology) diluted in blocking buffer. After washing, membranes were incubated with HRP-conjugated secondary antibodies (Amersham ECL) for 1 h at room temperature. Signal was detected using SuperSignal West Femto substrate (Thermo Scientific) and imaged on a Fujifilm LAS-4000.

### Xenograft models

Female athymic nude mice (Envigo RMS, Inc., Mount Comfort, Indiana), or NOD SCID (for H358 xenograft model) gamma female mice (The Jackson Laboratory, Bar Harbor, Maine), weighing 20 to 22 grams, were used for the studies. The animals were housed and provided free access to a standard diet and water. 5 × 10^6^ cells in a volume of 0.2 mL Hanks’ Balanced Salt solution (HBSS): Matrigel (Corning, Cat# 354234) (1:1) were implanted subcutaneously in the right flank of each animal. When the tumor volumes reached 200 − 300 mm^3^ the mice were randomized (*n* = 5-6 mice per group) based on tumor measurement and body weight using the multi-task block randomization tool. Each compound was initiated with oral administration (gavage) of either 0.2mL vehicle or compound for 28 days according to the experimental design. Statistical analysis results were summarized at Day 28 of treatment for the H358 and SW837 xenograft studies, and at Day 24 of treatment for the H1373 xenograft study. Tumor regrowth was monitored for an additional 10–18 days post last dose in the H358 studies. All studies and analysis were performed independent of the study investigators. Maximal tumor burden allowed was 2000 mm^3^. Tumor burden limits were monitored in accordance with institutional guidelines. In cases where tumor volumes exceeded the predefined threshold near study completion, animals were independently evaluated by institutional veterinary staff and confirmed to remain clinically well. Animals exceeding the tumor volume limit were removed from the study and euthanized following a final tumor measurement. Mice were housed in a 12 h day/night cycle, ambient temperature of 70–72 F and 30–40% humidity

### EL3187 PDX model

EL3187 tumor fragments were obtained from the Methodist Research Institute Biorepository. The PDX model was developed by subcutaneously implanting tumor fragments into the right rear flank of female 6-8 week old (20-22 gram) athymic nude-Foxn1nu feeder mice (from Envigo RMS, Inc., Mount Comfort, Indiana). Fresh tumors, passage 4, were cut into 10-15 mm^3^ fragments and placed into cold Gibco Hibernate Medium, and then the pooled tumor fragments were subcutaneously implanted into animals with a 10 g trochar needle. When the tumor size was approximately 200-300 mm^3^, the mice were randomized according to the experimental design. Groups were dosed by oral gavage with 0.2 mL of either vehicle or compound for 28 days. Tumor volumes were measured using calipers twice weekly. All studies and analysis were performed independent of the study investigators.

### Brain orthotopic model

The H358 cell line was cultured following ATCC protocols, and transduced with the lentivirus vector and polybrene after seeding overnight, following the manufacturer’s instructions. The plate was incubated at 37 °C and 5% CO_2_ for 24 h. After removing the virus-containing transduction medium, fresh growth medium was added, and the cells were incubated for an additional 48 h to allow protein expression. A stable cell line was then selected using puromycin antibiotics and evaluated for luciferase expression. Positive clones were cryopreserved and stored in liquid nitrogen until use.

Female athymic nude mice (Envigo RMS, Inc., Mount Comfort, Indiana) weighing 20-22 grams were housed in a 12-h light/dark cycle and provided with standard diet and water ad libitum. To establish the intracranial model, a needle was inserted 3 mm into the striatum and 2.5 × 10^5^ H358-Luc cells in 10 uL serum-free media was manually injected slowly. The health of the mice was monitored daily. Fifteen days after implantation the mice were then randomized into 2 groups (*n* = 9 mice per group) and were treated with either vehicle or LY3537982 at the 30mpk BID for 28 days. All studies and analysis were performed independently of the study investigators.

Tumor progression in the brain was assessed using the Perkin-Elmer In Vivo Imaging System (IVIS) weekly. Baseline bioluminescent imaging (BLI) was conducted on Day 15, 24 h prior to the initial dosing. For BLI, the animals were administered 150 mg/kg D-luciferin by intraperitoneal injection, followed by a 15-minute period allowing for distribution of the tracer. Immediately prior to imaging, the mice were anesthetized in an induction chamber with 1–5% isoflurane and maintained under anesthesia with a continuous flow of isoflurane and oxygen. Once anesthetized, the animals were transferred to the imaging chamber, where anesthesia was maintained by individual nosecones for imaging. Data analysis was performed using Living Image 4.7.2 software by drawing regions of interest over the head of the mouse or on the rear flank to serve as a non-specific background to generate background-normalized total flux values [photons/second] for each animal. As the total flux is proportional to tumor size, normalized total flux was used to evaluate tumor progression and treatment efficacy. Throughout the treatment period, the total flux and body weight were recorded weekly, and both measurements continued for 3 days following final dosing. The data were analyzed utilizing a data capture tool (StudyLog) and a proprietary analysis tool (ZEUS).

The statistical analysis of the total flux data began with a log transformation to equalize variance across time and treatment groups. The log total flux data were analyzed using a two-way repeated-measures analysis of variance by time and treatment, with the MIXED procedure in SAS (Version 9.3). The correlation model for the repeated measures is the Spatial Power model. Compound treated groups were compared to the control group at each time point. The MIXED procedure was also used separately for each treatment group to calculate adjusted means and standard errors at each time point. Both analyses account for the autocorrelation within each animal and the loss of data that occurs when animals with large tumors are removed from the study early. The adjusted means and standard errors were plotted for each treatment group versus time using Prism (GraphPad Software, LLC).

Efficacy was calculated at the end of the treatment. Data from earlier dates were used to calculate efficacy if more than half of the animals were lost in the vehicle group before treatment completion.

### CRISPR gene editing to knock in the secondary mutations

The H1373 cell line carrying secondary mutations were generated in house. The Cas 9 and single-guided RNA system were used. After DSB was generated, the homology-directed repair with a DNA template present was involved to repair the DSB to generate the desired sequences. The result is precise gene editing. WT: AGATATTCAC**CAT**TATAG H95D: AGATATCCAT**GAT**TACAG; H95Q: AGATATCCAT**CAG**TACAG; H95R: AGATATCCAT**CGT**TACAG; Y96C: AGATATCCATCAC**TGC**AG; Y96D: AGATATCCATCAC**GAT**AG.

The data were entered into GraphPad Prism, and the mean ± SEM for the treatment replicates (*n* = 3 biological replicates) was determined. The IC_50_ values were calculated by fitting the data at each drug concentration using the sigmoidal dose-response (variable slope) equation in GraphPad Prism.

For all studies, statistical analysis was performed using one-way ANOVA, unless indicated otherwise.

### Reporting summary

Further information on research design is available in the Nature Portfolio Reporting Summary linked to this article.

## Supplementary information

Supplementary Information

Reporting Summary

Transparent Peer Review file

## Source data

Source Data

## Data Availability

Eli Lilly and Company provides access to all individual data collected during the trial, after anonymization, with the exception of pharmacokinetic, genomic, or genetic data. Data are available to request 6 months after the indication studied has been approved in the US and EU and after primary publication acceptance, whichever is later. No expiration date of data requests is currently set once the data are made available. Access is provided after a proposal has been approved by an independent review committee identified for this purpose and after receipt of a signed data sharing agreement. Data and documents, including the study protocol, statistical analysis plan, clinical study report, and blank or annotated case report forms, will be provided in a secure data sharing environment. For details on submitting a request, see the instructions provided at www.vivli.org. All of the raw files of the mass spectrometry proteomics data have been deposited to ProteomeXchange via the jPOST repository (https://repository.jpostdb.org) with the identifier PXD070323 (https://repository.jpostdb.org/entry/JPST004169). The atomic coordinates and structure factors have been deposited in the Protein Data Bank under accession code 9N44. The remaining data are available within the Article, Supplementary Information or Source Data file. Source data are provided with this paper.
