# Supplementary material for: Characterization of KRASG12C inhibitor olomorasib single-agent and combination with activity in KRASG12C-mutant models
Source: Nat Commun. 2026 May 4;17:6001. doi: 10.1038/s41467-026-72650-y (PMC13346474; doi:10.1038/s41467-026-72650-y)
Supplement: Supplementary file 1 — Supplementary Information [file 41467_2026_72650_MOESM1_ESM.pdf]

Supplementary Information

Supplementary Notes

Synthesis of Olomorasib (LY3537982): Olomorasib was synthesized and characterized at Lilly Research Laboratories following procedures described in WO2023196959A1.

4-[(13aS)-10-Chloro-8-fluoro-6-oxo-2-prop-2-enoyl-1,3,4,12,13,13a-hexahydropyrazino[2,1-d] [1,5]benzoxazocin-9-yl]-2-amino-7-fluoro-benzothiophene-3-carbonitrile, M atropisomer (Olomorasib). HCl gas was bubbled for five minutes into an ice-cooled solution of *tert*-butyl (13aS)-9-[2-(*tert*-butoxycarbonylamino)-3-cyano-7-fluoro-benzothiophen-4-yl]-10-chloro-8-fluoro-6-oxo-1,3,4,12,13,13a-hexahydropyrazino[2, 1-d][1,5]benzoxazocine-2-carboxylate, M atropisomer (0.786 g, 1.17 mmol) in DCM (12 mL) and 2-propanol (12 mL). The reaction mixture was stirred at room temperature for five hours, then cooled in an ice bath. HCl gas was bubbled into the reaction mixture for five minutes. The reaction mixture was stirred at room temperature for 14 hours, then concentrated *in vacuo*. The residue was twice diluted with n-heptane and concentrated *in vacuo*. MTBE (50 mL) was added. The mixture was stirred at room temperature for ten minutes, then filtered to give the deprotected material as a dihydrochloride salt.

The dihydrochloride salt was dissolved in water (12 mL). 2-Methyltetrahydrofuran (12 mL) was added. A solution of potassium carbonate (0.81 g, 5.82 mmol) in water (12 mL) was added. The mixture was vigorously stirred while being cooled in an ice bath. Acryloyl chloride (0.10 mL, 1.28 mmol) in 2-methyltetrahydrofuran (5 mL) was added dropwise. After five minutes, diluted with brine and 2-methyltetrahydrofuran. The aqueous layer was extracted with 2-methyltetrahydrofuran (2x). The combined organic extracts were washed with water, dried over magnesium sulfate, filtered, and concentrated in vacuo. The crude was purified by silica gel flash chromatography (20-30% B in A; A:3: 1 EtOAc:hexanes; B: 4:1 EtOAc:MeOH) to give the title compound (0.409 g, 66%).

^{1}H NMR (400 MHz, DMSO-d6): δ 8.11 (s, 2H), 7.28 (d, J= 8.9 Hz, 1H), 7.20-7.11 (m, 2H), 6.91-6.73 (m, 1H), 6.22-6.12 (m, 1H), 5.76-5.72 (m, 1H), 4.48-3.90 (m, 6H), 3.51-3.40 (m, 1H), 3.36-3.00 (m, 2H), 2.24-2.08 (m, 1H), 1.82-1.62 (m, 1H).

^{13}C NMR (126 MHz, DMSO-d6) δ 166.55, 166.08, 164.68 (d, J = 28.9 Hz), 156.26 (d, J = 244.4 Hz), 155.17 (d, J = 239.5 Hz), 148.44, 138.00 (d, J = 5.3 Hz), 129.52 (d, J = 7.8 Hz), 128.23, 127.80, 127.63, 127.51, 127.14, 126.76 (d, J = 28.1 Hz), 119.19 (d, J = 3.1 Hz), 114.58 (d, J = 19.7 Hz), 113.93, 107.94 (d, J = 19.2 Hz), 77.24 (d, J = 3.1 Hz), 69.08 (d, J = 27.3 Hz), 51.42, 48.30 and 45.01, 44.02 and 40.73, 37.95 and 37.25, 30.04 and 29.30. (observed rotamers are noted with "and").

HRMS (ESI, m/z): calcd for $C_{25}H_{20}ClF_2N_4O_3S$ [M + H]+, 529.0907; found, 529.0882.

Specific NMR assignments were determined using HSQC experiments.

Supplementary Table 1

	KRAS-G12C with LY3537982
**Data collection**	
Space group	C 2
Cell dimensions	
a, *b*, *c* (Å)	85.503, 39.464, 53.222
α, β, γ (°)	90.00, 96.81, 90.00
Resolution (Å)	30.00-1.11 (1.21-1.14) *
CC(½)	0.998 (0.777)
R_{sym} or R_{merge}	7.5 (59.9)
$I / \sigma I$	14.9 (2.8)
Completeness (%)	75.8 (23.5)
Redundancy	6.6 (5.4)
**Refinement**	
Resolution (Å)	30.00-1.14
No. reflections	49674
R_{work} / R_{free}	0.163 / 0.189
No. atoms	
Protein	1322
Water	100
Other	41
B-factors	
Protein	13.07
Water	20.45
Other	8.06
R.m.s. deviations	
Bond lengths (Å)	0.010
Bond angles (°)	1.264
Ramachandran distribution	
Favored (%)	97.6
Allowed (%)	100
Outliers (%)	0

*Number of xtals for each structure should be noted in footnote. *Values in parentheses are for highest-resolution shell.

Supplementary Table 1. Data collection and refinement for olomorasib-KRAS^{G12C} co-crystal structure (molecular replacement).

Supplementary Table 2

Combination Index$_{50}$

Cell Line	Histology	Cetuximab	Gemcitabine	Erlotinib	Dasatinib	Everolimus	Abemaciclib	AKTi	Trametinib	ERKi	AurAi	Alpelisib	Erdafitinib	GPX4i	RMC-4550
H358	Lung	N/A	1.86	0.91	2.96	0.13	1.26	0.99	0.47	1.44	2.55	1.10	0.92	2.74	1.08
H1373	Lung	2.34	0.84	0.33	0.03	0.23	0.13	0.29	0.16	0.32	0.15	0.52	1.70	1.02	35.02
MIA-PaCa-2	Pancreas	N/A	1.58	0.87	0.88	0.50	1.53	1.14	0.35	0.61	1.00	0.87	0.74	1.52	0.73
H1792	Lung	1.35	N/A	0.97	0.04	0.02	0.10	0.36	0.54	0.46	0.27	0.21	0.03	1.02	0.10
H2030	Lung	1.07	1.68	0.34	0.11	0.01	0.47	0.15	0.29	0.31	0.19	0.18	0.47	0.28	0.39
HCC44	Lung	0.98	0.00	0.98	0.13	0.01	0.33	0.23	0.22	0.43	0.55	0.29	0.42	1.32	0.15
KYSE410	Oesophageal	0.82	0.21	0.26	0.43	0.99	0.14	0.54	0.22	1.01	0.11	0.68	0.74	1.71	0.12
SW837	Colon	0.74	2.57	0.51	0.30	0.64	1.04	0.78	0.27	0.56	2.31	0.33	1.54	0.90	0.34
SW1463	Colon	103.92	1.11	1.29	1278.13	14.15	2.17	4.75	0.18	57.62	0.21	1.67	4.09	8.94	1051.97

Supplementary Table 2. *In vitro* models were treated with the indicated compounds and combination index calculated. Green-colored cells indicate CI_{50} less than 0.5 (synergistic), pink-colored cells indicate CI_{50} 0.5 to 1.5 (additive), and red-colored cells indicate CI_{50} greater than 1.5 (antagonistic). n= 2 biological replicates.

Supplementary Table 3

	Treatment	Dose (mg/kg)	Dose Frequency	TGI (%) or Regression (-%)	P-value
**H358**	LY3537982	5	PO, BIDx28	39.7	0.083
	LY3537982	10	PO, BIDx28	74.2	<0.001
	LY3537982	30	PO, BIDx28	-28.1	<0.001
	**Treatment**	**Dose (mg/kg)**	**Dose Frequency**	**TGI (%) or Regression (-%)**	**P-value**
**H1373**	LY3537982	5	PO, BIDx28	-26.3	<.0001
	LY3537982	10	PO, BIDx28	-49	<.0001
	LY3537982	30	PO, BIDx28	NA	NA
	**Treatment**	**Dose (mg/kg)**	**Dose Frequency**	**TGI (%) or Regression (-%)**	**P-value**
**SW1463**	LY3537982	5	PO, BIDx28	48.8	0.0399
	LY3537982	10	PO, BIDx28	79.5	<.0001
	LY3537982	30	PO, BIDx28	98.8	<.0001
	**Treatment**	**Dose (mg/kg)**	**Dose Frequency**	**TGI (%) or Regression (-%)**	**P-value**
**MIA-Paca-2**	LY3537982	5	PO, BIDx28	-36.1	<0.001
	LY3537982	10	PO, BIDx28	-45.2	<0.001
	LY3537982	30	PO, BIDx28	-43.6	<0.001
	**Treatment**	**Dose (mg/kg)**	**Dose Frequency**	**TGI (%) or Regression (-%)**	**P-value**
**EL3187**	LY3537982	1	PO, BIDx28	89.2	0.008
	LY3537982	3	PO, BIDx28	-91	<0.001
	LY3537982	10	PO, BIDx28	-96	<0.001

Supplementary Table 3. Tumor growth inhibition for *in vivo* single agent olomorasib. *p<0.001, **p<0.0001

Supplementary Table 4

% Tumor Growth Inhibition or % Regression in Xenograft Models											
		Combination Agent, Dose, Dosing Schedule									
	LY3537982	RMC-4550	Alpelisib	Trametinib	AurAi	ERKi	Abemaciclib	Erlotinib	Cetuximab	Anti-PD1 (RMP1-14)	Anti-PDL1 (LSN3428712)
		30mpk	25mpk	0.3mpk	57mpk	100mpk	50mpk	25mpk	20mpk	250µg/ animal	500µg/ animal
Model	PO, QD	PO, QDx28	PO, QDx28	PO, QDx28	PO, BIDx28*	PO, QDx28	PO, QDx28	PO, QDx28	IP, BIWx4	IP,BIWx3	IP, Q7Dx3
EL3187	3mg/kg	-76.2	-73.8	-93.5	-88.6	-84	-96	-93.1	N/A	N/A	N/A
H1373	10mg/kg	-74	94.7	-68	-25.3	-70.2	-54.4	-48.9	N/A	N/A	N/A
H358	10mg/kg	-65.1	-32.3	-61.8	-55.3	-22.1	-61.8	-64	-34.6	N/A	N/A
MIA-PaCa-2	30mg/kg	-68.2	-57.4	-65.6	-61.1	-65.4	-59.8	-62.2	N/A	N/A	N/A
LU99	30mg/kg	-48.9	-27.1	-31.6	-62	-3.7	-37.4	98.9	N/A	N/A	N/A
SW837	10mg/kg	-34	N/A	98.4	-6.4	-30	-18.2	-27.8	-37.4	N/A	N/A
CT26	30mg/kg	N/A	N/A	N/A	N/A	N/A	N/A	N/A	N/A	-19.4	-33.9

Supplementary Table 4. *In vivo* tumor growth inhibition of olomorasib in combination with anti-cancer treatments. *H1373 - BIDx3, QDx25; SW1463 - BIDx12, QDx16; Statistical combination effect: green fill – no combination effect, yellow fill – additive, red fill – greater than additive

Supplementary Table 5

	Treatment	Dose (mg/kg)	Dose Frequency	TGI (%) or Regression (-%)	P-value
H358	Vehicle		PO, QDx28	NA	
	LY3537982	10	PO, QDx28	74.9	<.0001
	RMC-4550	30	PO, QDx28	71.7	<.0001
	Alpelisib	25	PO, QDx28	78.4	<.0001
	AurA	57	PO, BIDx28	77.6	<.0001
	ERK	100	PO, QDx28	71.1	<.0001
	Abemaciclib	50	PO, QDx28	77	<.0001
	LY3537982 + RMC-4550	10	PO, QDx28	-65.1	<.0001
		30	PO, QDx28		
	LY3537982 + Alpelisib	10	PO, QDx28	-32.3	<.0001
		25	PO, QDx28		
	LY3537982 + AurA	10	PO, QDx28	-55.3	<.0001
		57	PO, BIDx28		
	LY3537982 + ERK	10	PO, QDx28	-22.1	<.0001
		100	PO, QDx28		
	LY3537982 + Abemaciclib	10	PO, QDx28	-61.8	<.0001
		50	PO, QDx28		
	**Treatment**	**Dose (mg/kg)**	**Dose Frequency**	**TGI (%) or Regression (-%)**	**p-value**
SW837	Vehicle		PO, QDx28		
	LY3537982	10	PO, QDx28	70.6	0.006
	Cetuximab	20	IP, BIWx4	53.9	0.0471
	LY3537982 + Cetuximab	10 + 20	PO, QDx28 + IP, BIWx4	-37.4	<.0001
	**Treatment**	**Dose (mg/kg)**	**Dose Frequency**	**TGI(%) or Regression(-%)**	**P-value**
CT26 Syngeneic	LY3537982	30	PO, QDx21	88.4	0.0003
	LSN3428712(PDL1)	500ug/animal	IP, Q7Dx3	36.1	0.3728
	PD1(RMP1-14)	250ug/animal	IP,BIWx3	70.7	0.0263
	LY3537982 + LSN3428712	30 + 500ug	PO, QDx21 + IP, Q7Dx3	-33.9	<.0001
	LY3537982 + PD1(RMP1-14)	30 + 250ug	PO, QDx21 + IP,BIWx3	-19.4	<.0001

Supplementary Table 5. *In vivo* tumor growth inhibition of olomorasib in combination with anti-cancer treatments. *p<0.001, **p<0.0001

Supplementary Figure 1

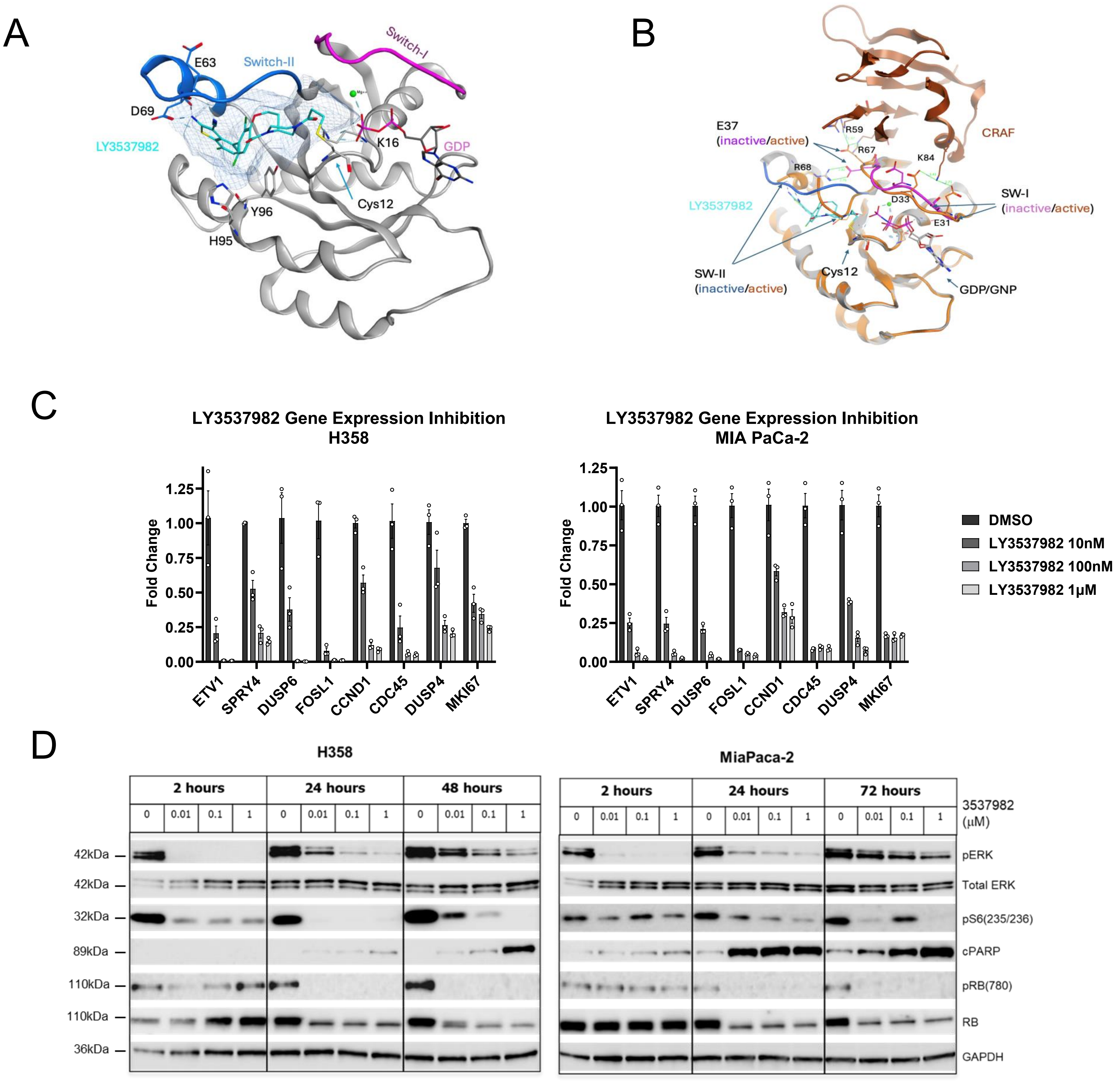

Supplementary Figure 1. Structural alignment of KRAS^{G12C} with LY3537982 and related gene expression (**A**) Co-crystal structure of olomorasib with KRAS^{G12C}. (**B**) Structural alignment of KRAS-G12C in its inactive form with covalently bound LY3537982 (gray, with SW-I in pink and SW-II in deep blue) and the active form of KRAS (orange, with SW-II partially visible due to its flexibility) in complex with GNP and CRAF (blue) (PDB ID: 6XHB). Olomoraisb (LY3537982) binds covalently to Cysteine 12, locking SW-I/SW-II into an inactive conformation and blocking interaction with CRAF. This binding induces a salt bridge formation between R68 of the SW-II loop and E37, which are critical residues for interaction with R59/R67/CRAF. Additionally, conformational changes in E31 and D33 obstruct the interaction with K84/CRAF. (**C**) Gene expression (n=3 experimental replicates) and (**D**) Western blot analysis in cells treated with olomorasib. Western blots are representative of at least 3 independent experiments

Supplementary Figure 2

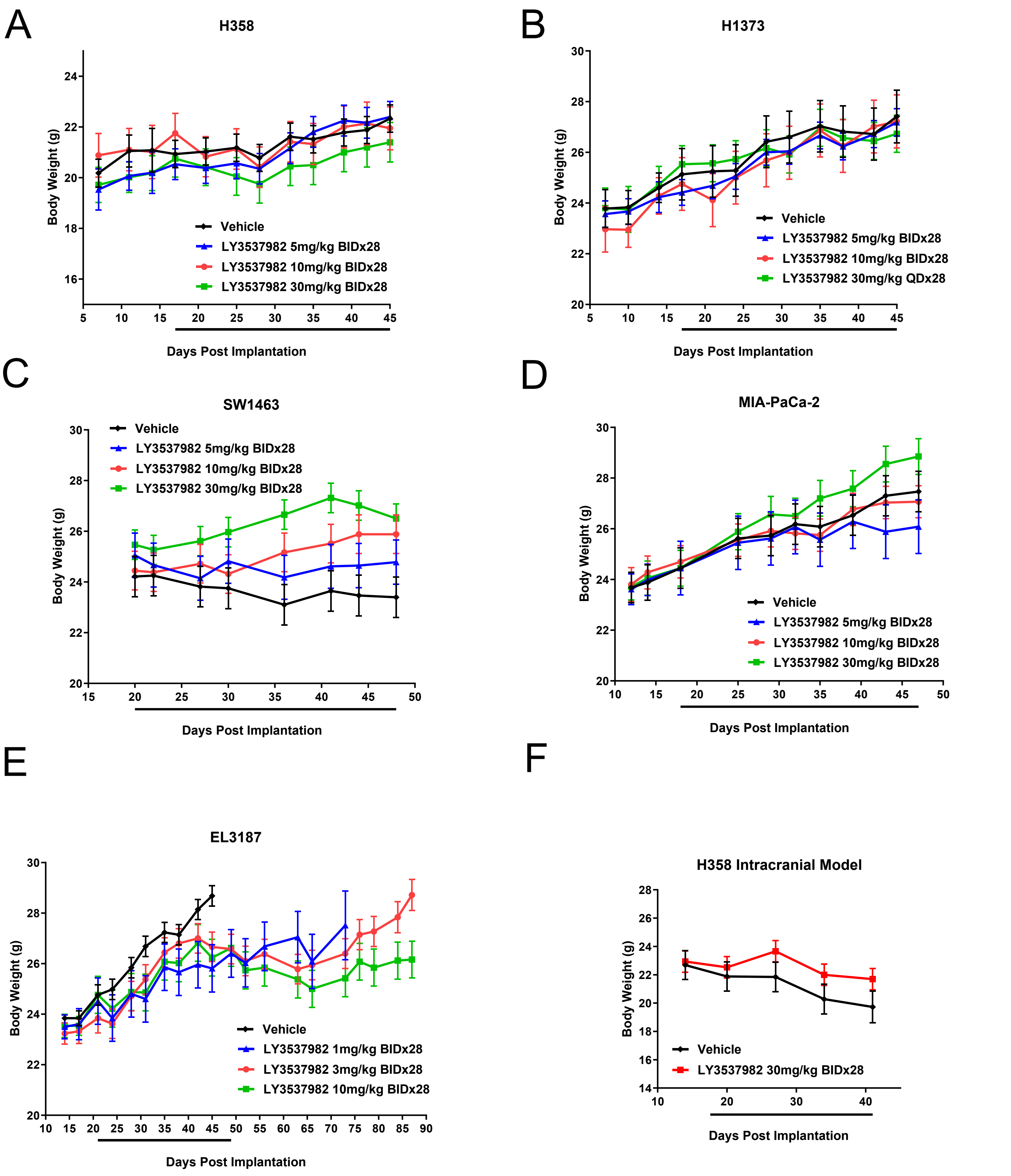

Supplementary Figure 2. Single-agent olomorasib is well-tolerated in xenograft models. (**A-F**) Tumor-bearing mice were treated with the indicated dose of olomorasib alone and body weight was measured at the indicated time points. (A-D) n = 6. (E) n = 5. (F) n = 9. Data represent mean ± SEM.

Supplementary Figure 3

A

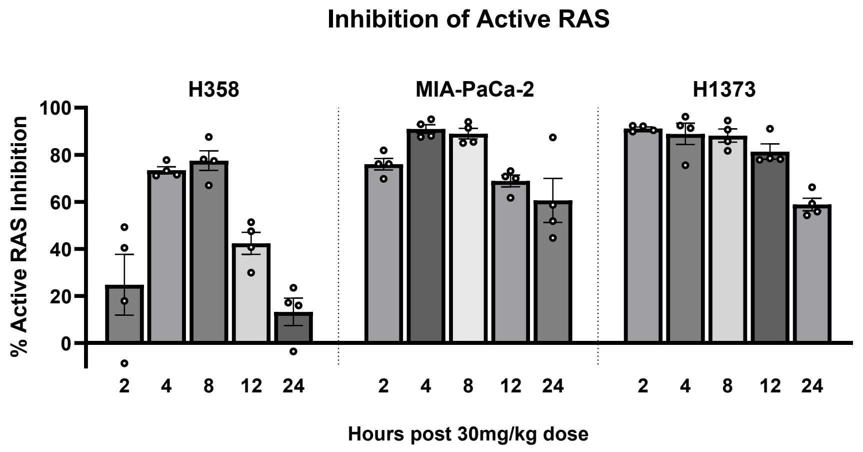

B

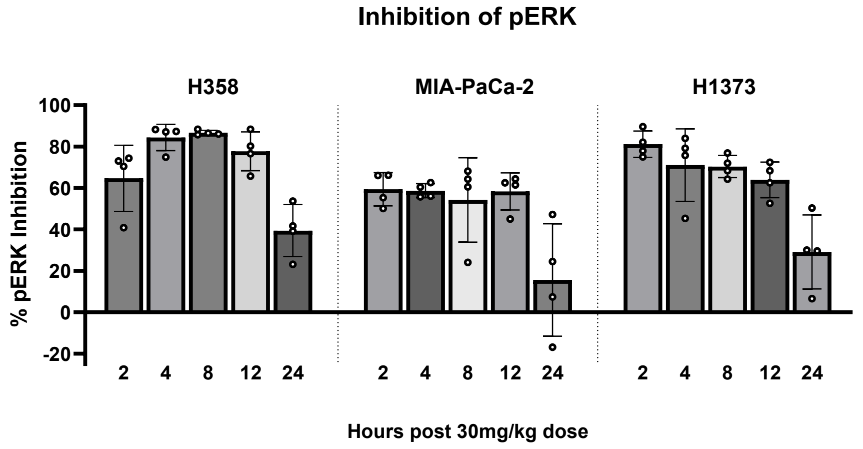

Supplementary Figure 3. Cancer cells were treated with olomorasib and (**A**) active RAS and (**B**) pERK activity measured. One-way ANVOA was used. Statistical analysis are presented in the Source Data. Data represent mean ± SEM, n = 4 biological replicates.

Supplementary Figure 4

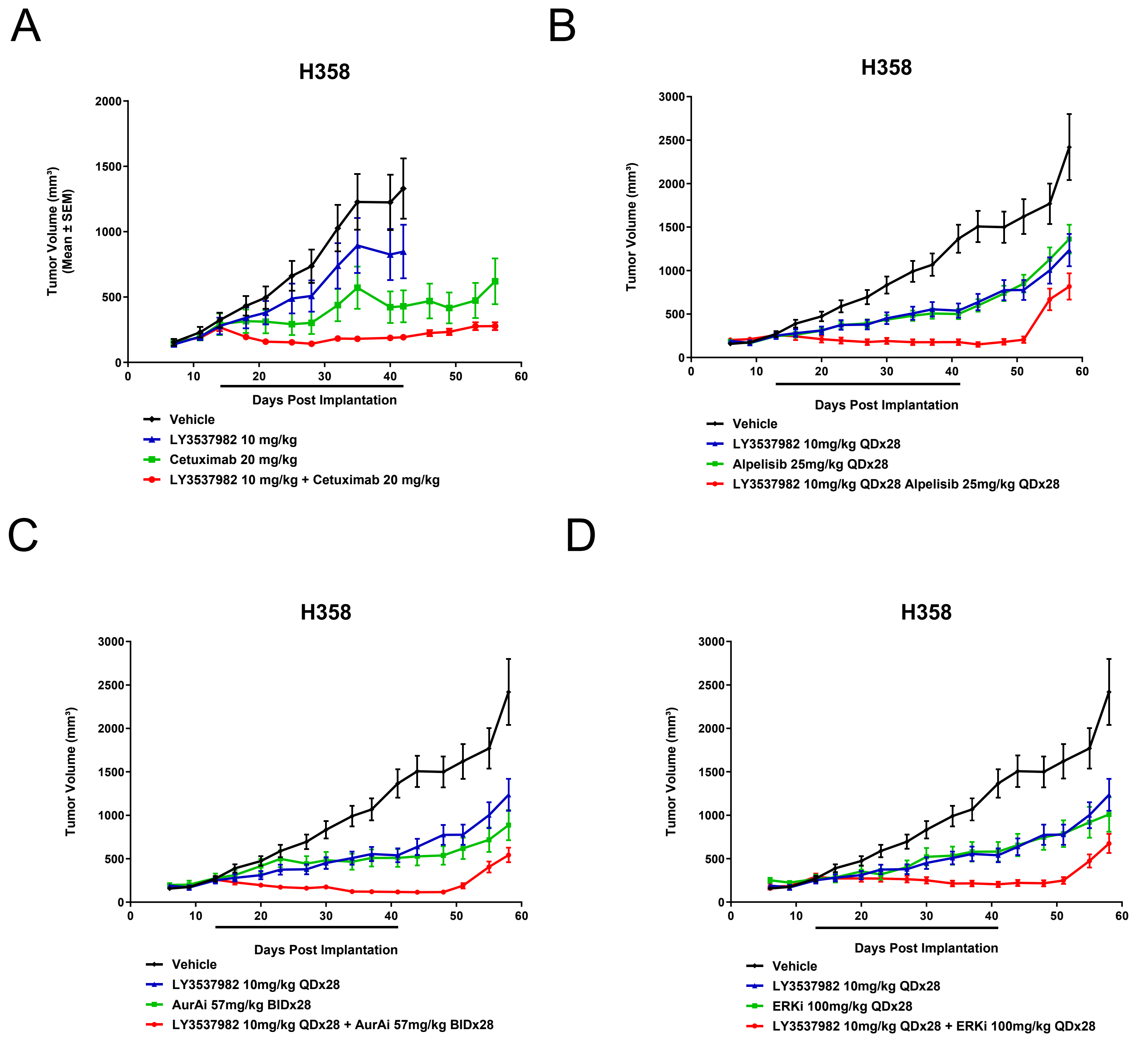

Supplementary Figure 4. Indicated cell line-derived xenograft (CDX) cancer models were implanted and treated with olomorasib alone and in combination with (**A**) cetuximab, (**B**) alpelisib (**C**) Aurora A inhibitor, and (**D**) ERK inhibitor. Tumor volume was measured at indicated time points. Black line represents treatment period. Statistical analysis is represented in **Supplementary Table 2**. mpk = mg/kg. Data represent mean ± SEM, n = 5-6 mice.

Supplementary Figure 5

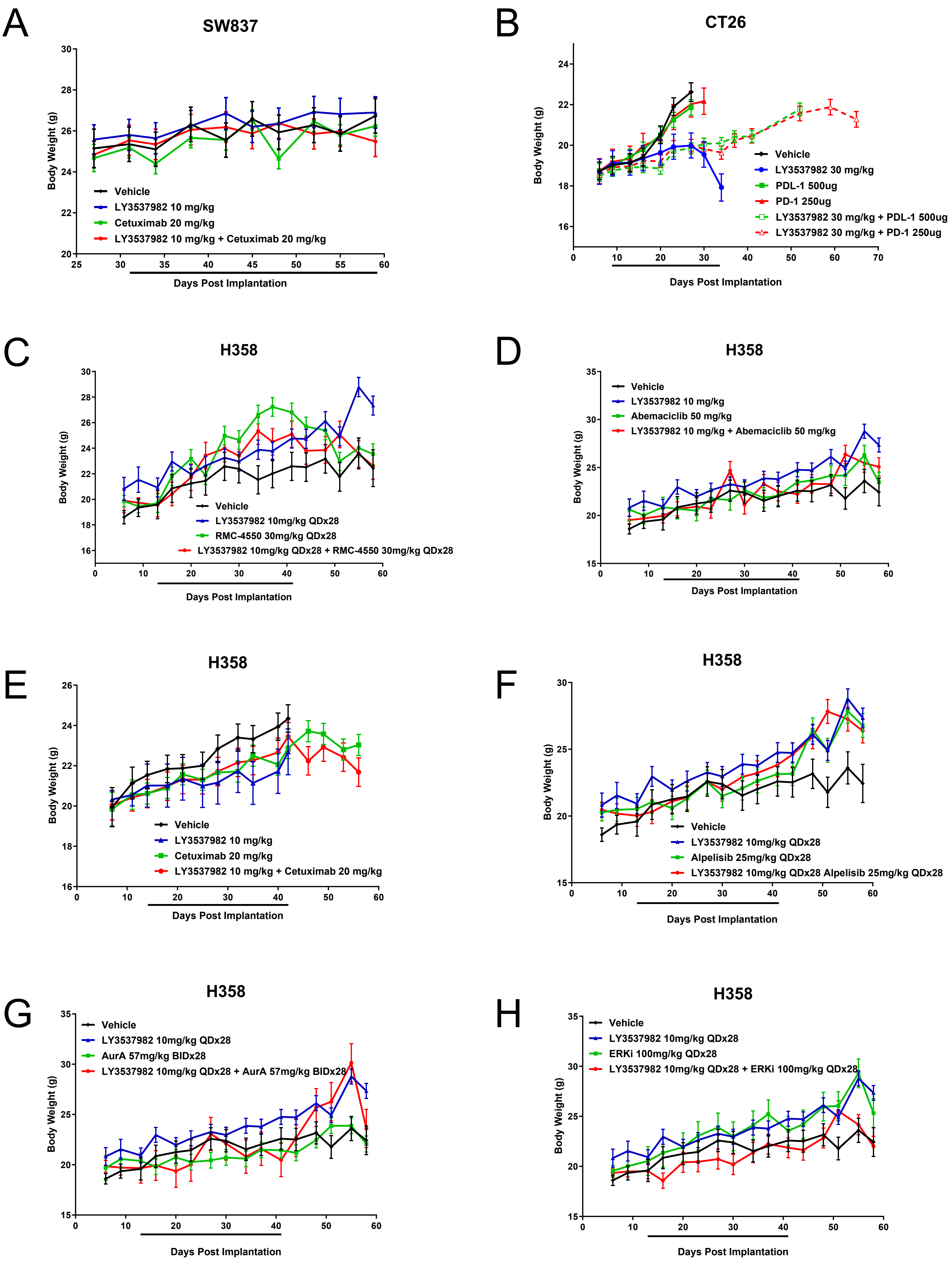

Supplementary Figure 5. Olomorasib in combination with additional therapeutics is well-tolerated in xenograft models. (**A-H**) Mice were treated with olomorasib and the indicated anti-cancer treatments and body weight was measured at the indicated time points. (A) n = 5-6 mice, (B) n = 10 mice and (C-H) n = 5-6 mice Data represent mean ± SEM.

Supplementary Figure 6

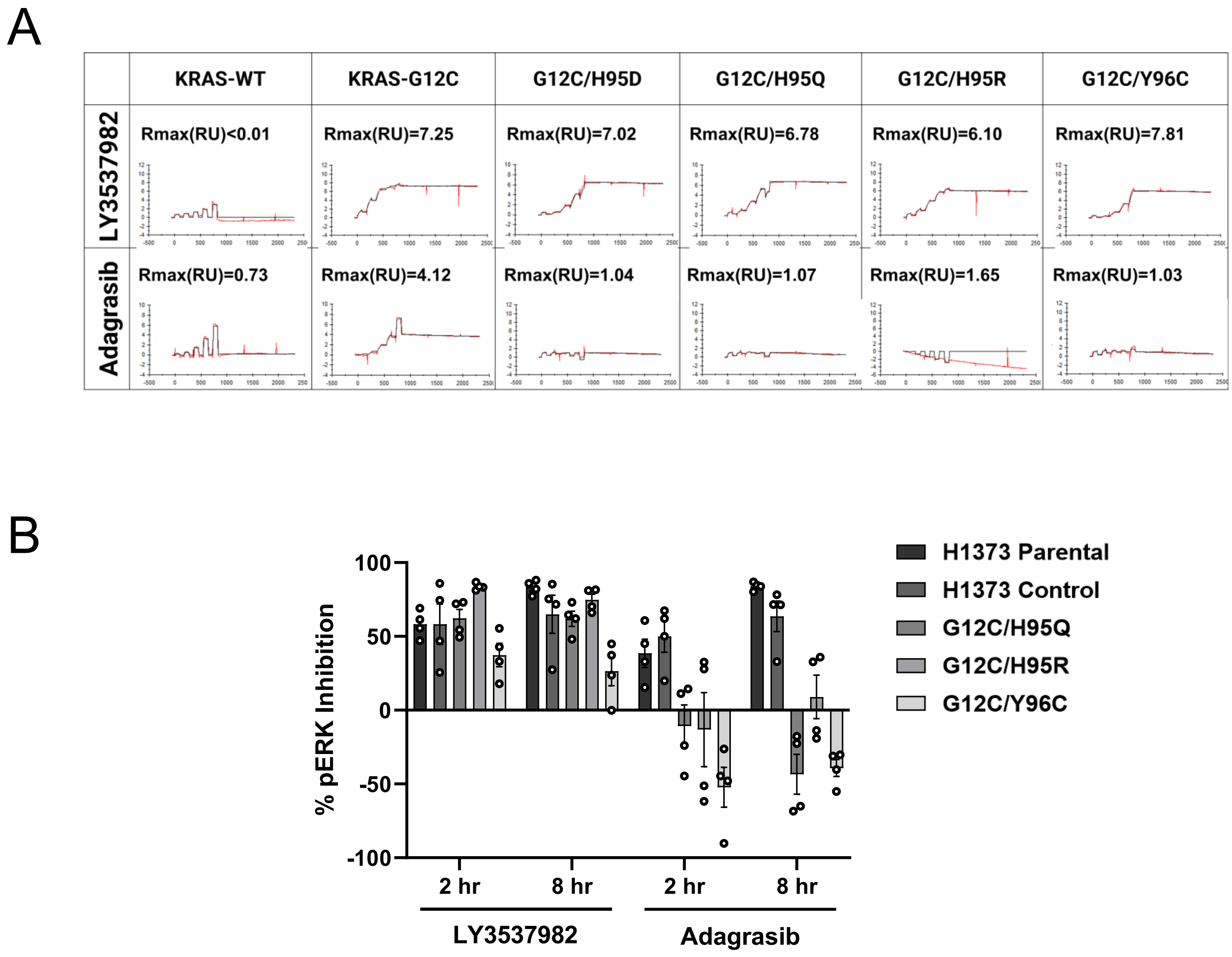

Supplementary Figure 6. Olomorasib binds and inhibits KRAS^{G12C} harboring second-site mutations with high affinity. (A) Surface plasmon resonance (SPR) and **(B)** %pERK inhibition to determine olomorasib (LY35379832) ability to bind to and inhibit, respectively, clinically relevant KRAS^{G12C} second-site mutations compared to adagrasib. Data represent mean ± SEM, n = 4.

Supplementary Figure 7

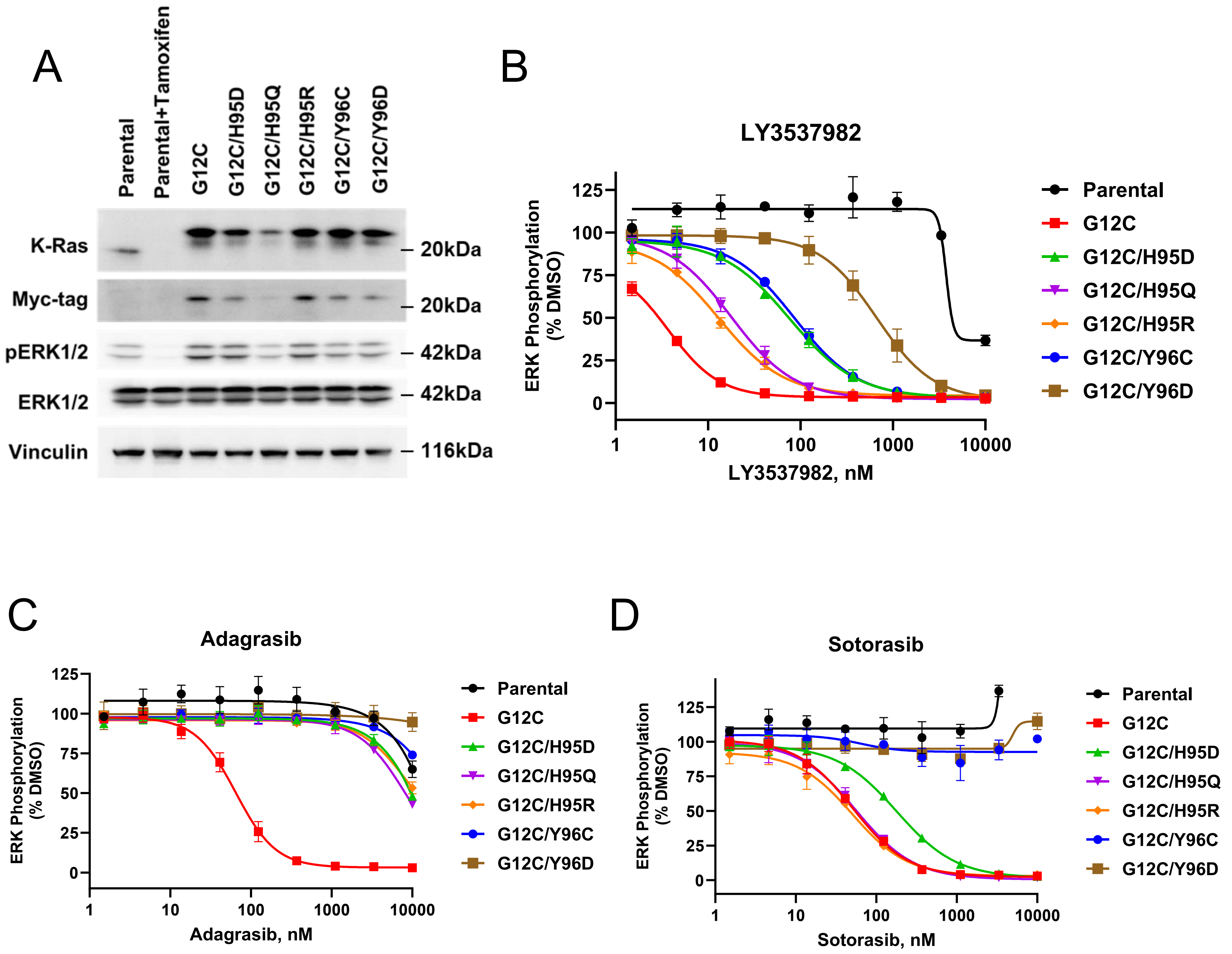

Supplementary Figure 7. Olomorasib retains activity in a RASless MEF model expressing KRAS^{G12C} second-site mutants that confer resistance to adagrasib and sotorasib. (A) Western blot analysis of cells expressing clinically relevant KRAS^{G12C} second site mutations. Cells expressing the indicated KRAS^{G12C} second-site mutations were treated with either **(B)** olomorasib, **(C)** adagrasib or **(D)** sotorasib and ERK phosphorylation assay was performed. (B-D) Data represent mean ± SEM, n = 3 experimental replicates unless otherwise stated.

Supplementary Figure 8

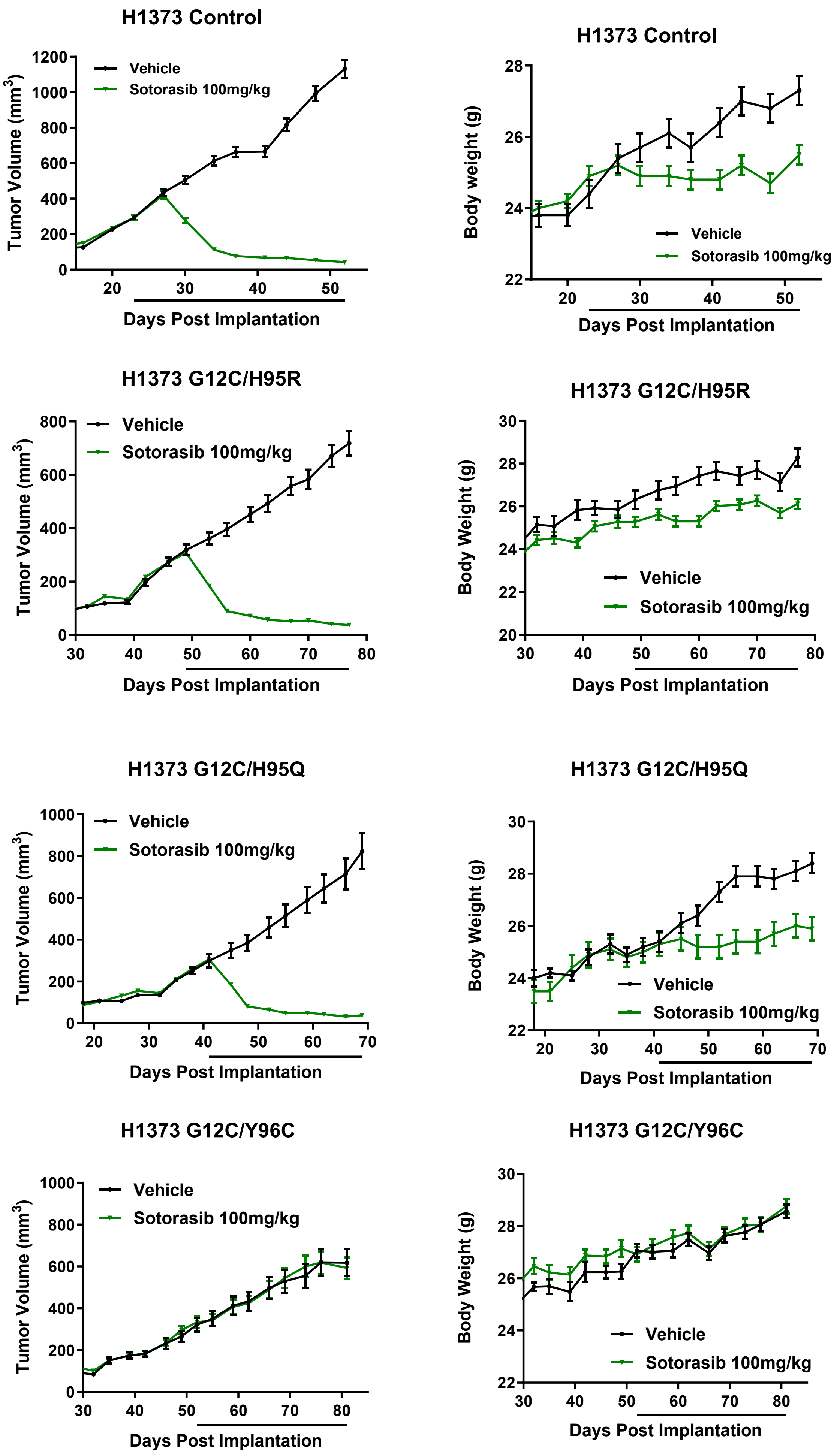

Supplementary Figure 8. Tumor Volume and Body Weight for KRAS^{G12C} second site mutations treated with sotorasib. Cancer cells expressing the indicated KRAS^{G12C} second site mutations were implanted into mice as previously described and treated. Tumor volume and body weight changes were measured at the indicated times. Data represent mean ± SEM, n = 5 mice.

Supplementary Figure 9

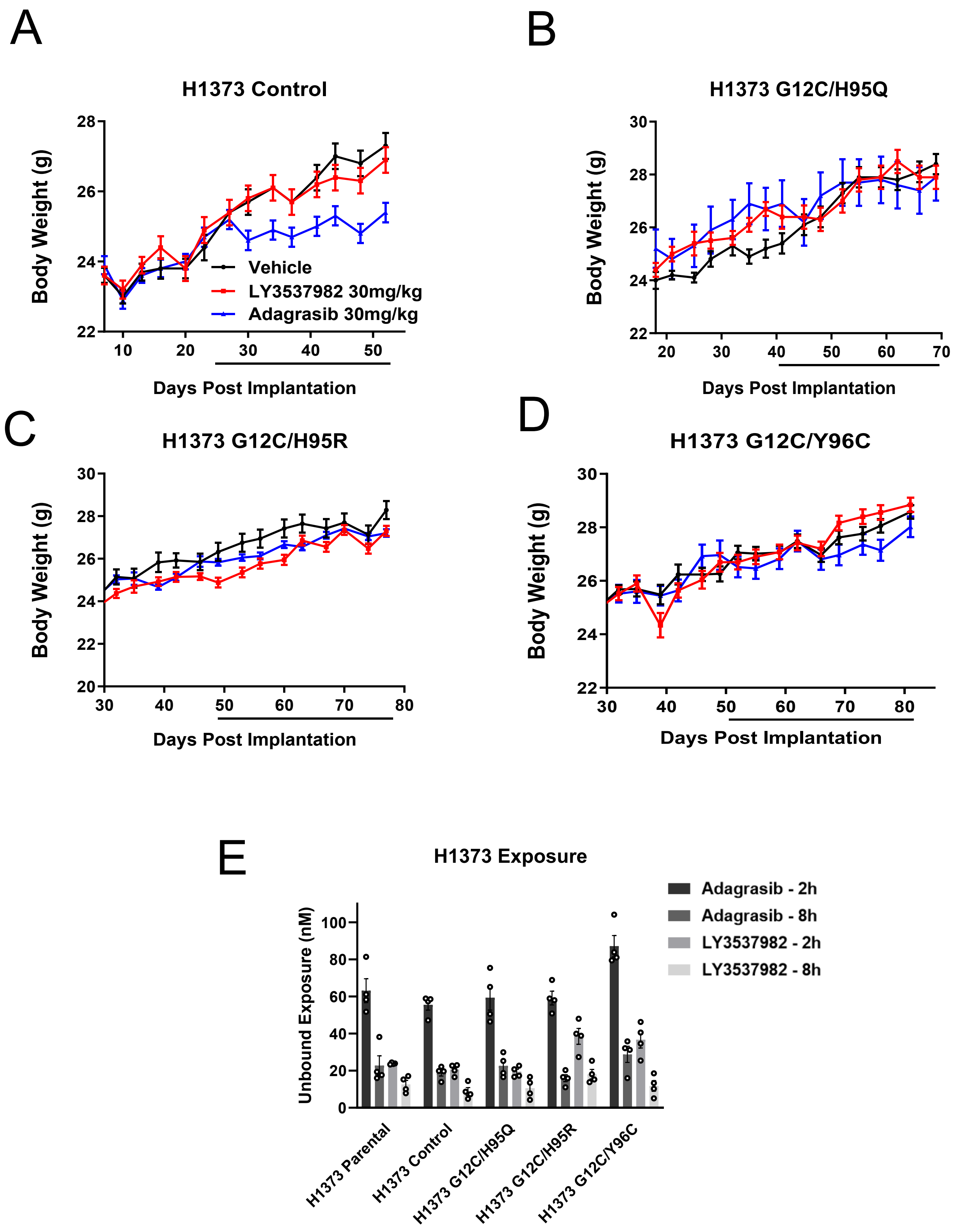

Supplementary Figure 9. Body weight and exposure for KRAS^{G12C} CRISPR knock-in models of second site mutations. Cancer cells expressing the indicated KRAS^{G12C} second site mutations were implanted into mice as previously described and treated. (**A-D**) Body Weight changes were measured at the indicated times, n = 5 mice. **(E)** Unbound drug exposure was measured in mice harboring the indicated KRAS^{G12C} second-site mutation. Data represent mean ± SEM, n = 4 biological replicates unless otherwise stated.

Supplementary Figure 10

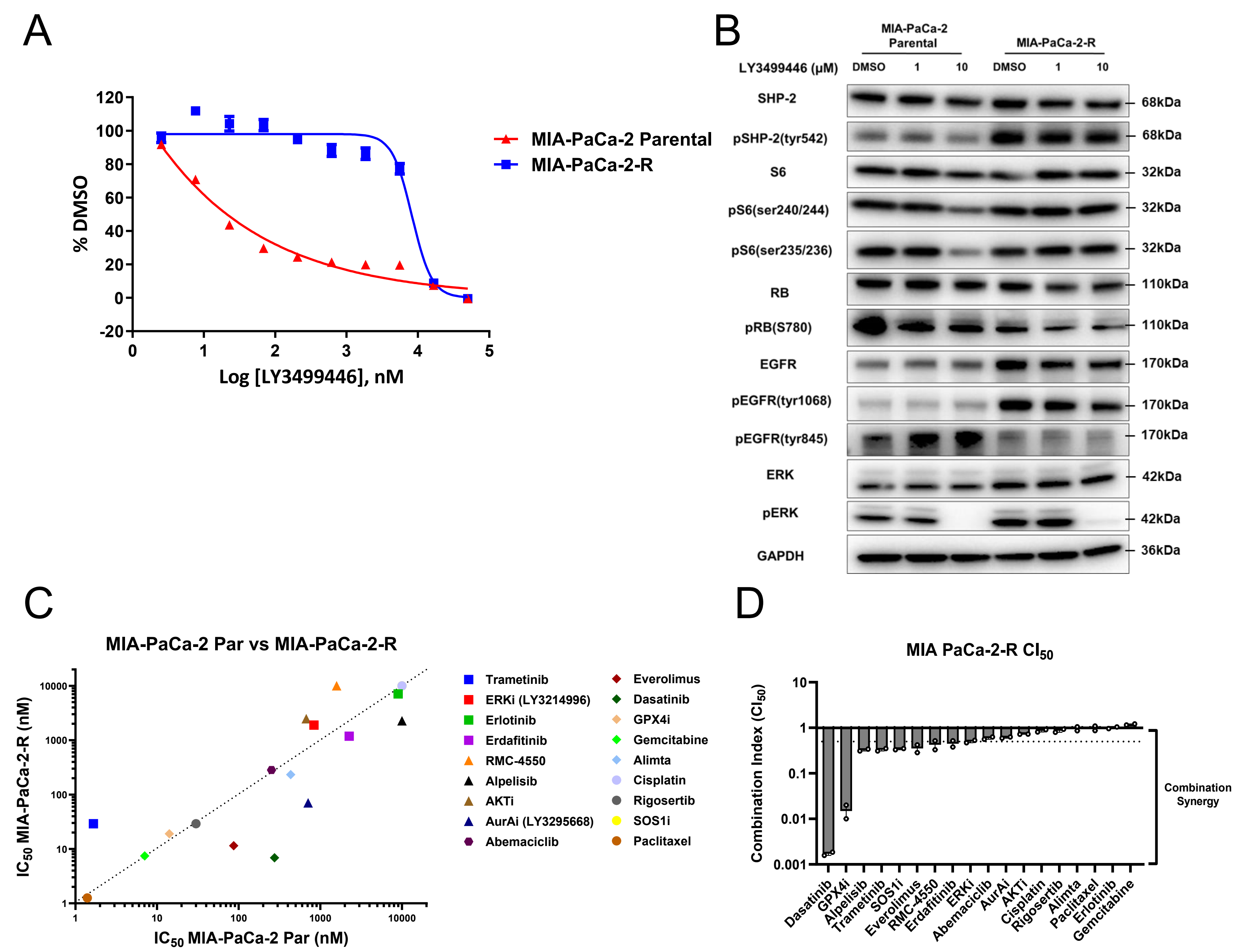

Supplementary Figure 10. Resistance to KRAS^{G12C} inhibitors induces targetable acquired dependence in cancer cells. (A) MIA-PaCa-2 parental (n=6 biological replicates) and resistant (n=3 biological replicates) cells were treated LY3499446, an analog of olomorasib (**NCT04165031),** until resistance was observed. **(B)** Western blot against indicated proteins was performed, and representative of at least 3 independent experiments. LY3499446-resistant cells were treated with a panel of anti-cancer drugs alone or in combination with LY3537982 and **(C)** IC_{50} (data presented is representative of replicate data) and **(D)** CI_{50} were measured. (C and D) n=2 biological replicates. Data available in Source File
