# Supplementary material for: Characterization of KRASG12C inhibitor olomorasib single-agent and combination with activity in KRASG12C-mutant models
Source: Nat Commun. 2026 May 4;17:6001. doi: 10.1038/s41467-026-72650-y (PMC13346474; doi:10.1038/s41467-026-72650-y)
Supplement: Supplementary file 2 — Reporting Summary [file 41467_2026_72650_MOESM2_ESM.docx]

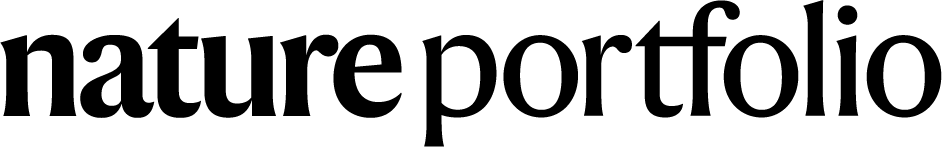
Corresponding author(s): Xueqian Gong

Last updated by author(s): Nov 19, 2025

Reporting Summary

Nature Portfolio wishes to improve the reproducibility of the work that we publish. This form provides structure for consistency and transparency in reporting. For further information on Nature Portfolio policies, see our Editorial Policies and the Editorial Policy Checklist.

Please do not complete any field with "not applicable" or n/a. Refer to the help text for what text to use if an item is not relevant to your study. For final submission: please carefully check your responses for accuracy; you will not be able to make changes later.

## Statistics

For all statistical analyses, confirm that the following items are present in the figure legend, table legend, main text, or Methods section.

n/a


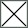

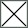

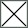


Confirmed


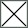
 The exact sample size (*n*) for each experimental group/condition, given as a discrete number and unit of measurement


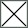
 A statement on whether measurements were taken from distinct samples or whether the same sample was measured repeatedly The statistical test(s) used AND whether they are one- or two-sided

*Only common tests should be described solely by name; describe more complex techniques in the Methods section.*


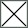
 A description of all covariates tested


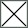
 A description of any assumptions or corrections, such as tests of normality and adjustment for multiple comparisons

A full description of the statistical parameters including central tendency (e.g. means) or other basic estimates (e.g. regression coefficient) AND variation (e.g. standard deviation) or associated estimates of uncertainty (e.g. confidence intervals)

For null hypothesis testing, the test statistic (e.g. *F*, *t*, *r*) with confidence intervals, effect sizes, degrees of freedom and *P* value noted

1

nature portfolio | reporting summary

*April 2023*

*Give P values as exact values whenever suitable.*

For Bayesian analysis, information on the choice of priors and Markov chain Monte Carlo settings

For hierarchical and complex designs, identification of the appropriate level for tests and full reporting of outcomes Estimates of effect sizes (e.g. Cohen's *d*, Pearson's *r*), indicating how they were calculated

*Our web collection on statistics for biologists contains articles on many of the points above.*

## Software and code

*Provide a description of all commercial, open source and custom code used to collect the data in this study, specifying the version used OR state that no software was used.*

Policy information about availability of computer code Data collection

Data analysis

*Provide a description of all commercial, open source and custom code used to analyse the data in this study, specifying the version used OR state that no software was used.*

For manuscripts utilizing custom algorithms or software that are central to the research but not yet described in published literature, software must be made available to editors and reviewers. We strongly encourage code deposition in a community repository (e.g. GitHub). See the Nature Portfolio guidelines for submitting code & software for further information.

## Data

Policy information about availability of data

All manuscripts must include a data availability statement. This statement should provide the following information, where applicable:

- Accession codes, unique identifiers, or web links for publicly available datasets
- A description of any restrictions on data availability
- For clinical datasets or third party data, please ensure that the statement adheres to our policy

All data supporting the findings of this study are available within the paper and its Supplementary Information.

2

nature portfolio | reporting summary

*April 2023*

## Research involving human participants, their data, or biological material

Policy information about studies with human participants or human data. See also policy information about sex, gender (identity/presentation), and sexual orientation and race, ethnicity and racism.

Reporting on sex and gender

*Use the terms sex (biological attribute) and gender (shaped by social and cultural circumstances) carefully in order to avoid confusing both terms. Indicate if findings apply to only one sex or gender; describe whether sex and gender were considered in study design; whether sex and/or gender was determined based on self-reporting or assigned and methods used.*

*Provide in the source data disaggregated sex and gender data, where this information has been collected, and if consent has been obtained for sharing of individual-level data; provide overall numbers in this Reporting Summary. Please state if this information has not been collected.*

*Report sex- and gender-based analyses where performed, justify reasons for lack of sex- and gender-based analysis.*

Reporting on race, ethnicity, or other socially relevant groupings

*Please specify the socially constructed or socially relevant categorization variable(s) used in your manuscript and explain why they were used. Please note that such variables should not be used as proxies for other socially constructed/relevant variables (for example, race or ethnicity should not be used as a proxy for socioeconomic status).*

*Provide clear definitions of the relevant terms used, how they were provided (by the participants/respondents, the researchers, or third parties), and the method(s) used to classify people into the different categories (e.g. self-report, census or administrative data, social media data, etc.)*

*Please provide details about how you controlled for confounding variables in your analyses.*

Population characteristics

*Describe the covariate-relevant population characteristics of the human research participants (e.g. age, genotypic information, past and current diagnosis and treatment categories). If you filled out the behavioural & social sciences study design questions and have nothing to add here, write "See above."*

Recruitment

*Describe how participants were recruited. Outline any potential self-selection bias or other biases that may be present and how these are likely to impact results.*

Ethics oversight

*Identify the organization(s) that approved the study protocol.*

Note that full information on the approval of the study protocol must also be provided in the manuscript.

# Field-specific reporting

Please select the one below that is the best fit for your research. If you are not sure, read the appropriate sections before making your selection.


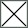
 Life sciences
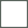
 Behavioural & social sciences
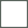
 Ecological, evolutionary & environmental sciences

For a reference copy of the document with all sections, see nature.com/documents/nr-reporting-summary-flat.pdf

# Life sciences study design

The sample size for our in vivo study was determined based on alignment with similar studies and the anticipated substantial effect size from preliminary data and existing literature.

All studies must disclose on these points even when the disclosure is negative. Sample size

Data exclusions

No data was excluded from analysis.

Replication

Replicates were performed in triplicate, unless otherwise stated.

Randomization

For in vivo studies, 15 days post-implantation mice were randomized into treatment groups.

Blinding

Randomization and data analysis was was performed independent of the primary researchers.

# Reporting for specific materials, systems and methods

We require information from authors about some types of materials, experimental systems and methods used in many studies. Here, indicate whether each material, system or method listed is relevant to your study. If you are not sure if a list item applies to your research, read the appropriate section before selecting a response.

3

nature portfolio | reporting summary

*April 2023*

Materials & experimental systems Methods


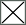

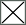

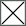

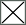


n/a Involved in the study Antibodies Eukaryotic cell lines

Palaeontology and archaeology Animals and other organisms Clinical data

Dual use research of concern

Plants


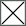

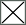

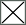


n/a Involved in the study

ChIP-seq

Flow cytometry

MRI-based neuroimaging

## Antibodies

Antibodies used

SHP2 (CST, 3397) pSHP2 (CST, 3751) S6 (CST, 2217) pS6 (CST, 4858) Rb (CST, 9309) pRb (CST, 8516) EGFR (CST, 4267) pEGFR-tyr1068

(CST, 3777) pEGFR-tyr845 (CST, 2231) ERK (CST, 4695) pERK (CST, 4370) GAPDH (CST, 2118) KRAS (CST, 33197) myc-tag (CST, 2276) Vinculin (CST, 4650) cPARP (CST, 5625) active KRAS (Sigma, WH0003845M1) RAS-GTP (CST, 8821) pMEK (CST, 9154) panRAS (CST, 3965) MEK (CST, 8727)

Validation

All antibodies were previously validated by the manufacturer

## Eukaryotic cell lines

Policy information about cell lines and Sex and Gender in Research Cell line source(s)

All cell lines were obtained from ATCC, unless indicated otherwise (H358: CRL-5807; CT26: CRL-2638; SW837: CCL-235; SW1463: CCL-234; MIA PACA-2: CRM-CRL-1420; H1373: CRL-5866; H1792: CRL-5895; H2030: CRL-5914; H2122: CRL-5985; SW1573: CRL-2170), (DMZ – KYSE-410: ACC 381; HCC-44: ACC-534), with the following exceptions: LXFA-983L (oncotest, Charles River Laboratories, MA); EL3187 (developed at Eli Lilly and Company, derived from a PDX model); LU99 (Japanese Collection of Research Bioresources Cell Bank)

Authentication

9-marker STR profile was used for cell line validation

Mycoplasma contamination Commonly misidentified lines

All cell lines tested negative for mycoplasma contamination

*Name any commonly misidentified cell lines used in the study and provide a rationale for their use.*

(See ICLAC register)

## Animals and other research organisms

Policy information about studies involving animals; ARRIVE guidelines recommended for reporting animal research, and Sex and Gender in Research

Laboratory animals

Female athymic nude mice or NOD SCID (H358 xenograft model);

Wild animals

*Provide details on animals observed in or captured in the field; report species and age where possible. Describe how animals were caught and transported and what happened to captive animals after the study (if killed, explain why and describe method; if released, say where and when) OR state that the study did not involve wild animals.*

Reporting on sex

*Indicate if findings apply to only one sex; describe whether sex was considered in study design, methods used for assigning sex. Provide data disaggregated for sex where this information has been collected in the source data as appropriate; provide overall numbers in this Reporting Summary. Please state if this information has not been collected. Report sex-based analyses where performed, justify reasons for lack of sex-based analysis.*

Field-collected samples

*For laboratory work with field-collected samples, describe all relevant parameters such as housing, maintenance, temperature, photoperiod and end-of-experiment protocol OR state that the study did not involve samples collected from the field.*

Ethics oversight

*Identify the organization(s) that approved or provided guidance on the study protocol, OR state that no ethical approval or guidance was required and explain why not.*

Note that full information on the approval of the study protocol must also be provided in the manuscript.

4

nature portfolio | reporting summary

*April 2023*

## Plants

Seed stocks

*Report on the source of all seed stocks or other plant material used. If applicable, state the seed stock centre and catalogue number. If plant specimens were collected from the field, describe the collection location, date and sampling procedures.*

*Describe the methods by which all novel plant genotypes were produced. This includes those generated by transgenic approaches, gene editing, chemical/radiation-based mutagenesis and hybridization. For transgenic lines, describe the transformation method, the number of independent lines analyzed and the generation upon which experiments were performed. For gene-edited lines, describe the editor used, the endogenous sequence targeted for editing, the targeting guide RNA sequence (if applicable) and how the editor was applied.*

*Describe any authentication procedures for each seed stock used or novel genotype generated. Describe any experiments used to*

*assess the effect of a mutation and, where applicable, how potential secondary effects (e.g. second site T-DNA insertions, mosiacism, off-target gene editing) were examined.*

Novel plant genotypes

Authentication


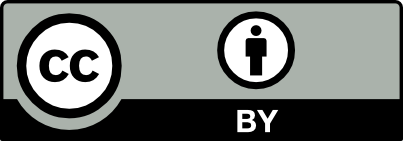
This checklist template is licensed under a Creative Commons Attribution 4.0 International License, which permits use, sharing, adaptation, distribution and reproduction in any medium or format, as long as you give appropriate credit to the original author(s) and the source, provide a link to the Creative Commons license, and indicate if changes were made. The images or other third party material in this article are included in the article's Creative Commons license, unless indicated otherwise in a credit line to the material. If material is not included in the article's Creative Commons license and your intended use is not permitted by statutory regulation or exceeds the permitted use, you will need to obtain permission directly from the copyright holder. To view a copy of this license, visit <http://creativecommons.org/licenses/by/4.0/>
