# Supplementary material for: Characterization of KRASG12C inhibitor olomorasib single-agent and combination with activity in KRASG12C-mutant models
Source: Nat Commun. 2026 May 4;17:6001. doi: 10.1038/s41467-026-72650-y (PMC13346474; doi:10.1038/s41467-026-72650-y)
Supplement: Supplementary file 3 — Transparent Peer Review file [file 41467_2026_72650_MOESM3_ESM.pdf]

nature portfolio

Peer Review File

Characterisation of KRAS-G12C inhibitor olomorasib single-agent and combination with activity in KRAS G12C-mutant models

Corresponding Author: Dr Xueqian Gong

This file contains all reviewer reports in order by version, followed by all author rebuttals in order by version.

Version 0:

Reviewer comments:

Reviewer #1

(Remarks to the Author)
Peng et al characterise olomorasib, a second generation covalent KRAS-G12C inhibitor, using biochemical, cellular and in vivo studies. They also test several combinations in vitro and in vivo and show that olomorasib is active in the presence of clinically relevant mutations that confer resistant to adagrasib. This is a well performed and timely study considering the progress of olomorasib in clinical studies.

The authors describe olomorasib as a second generation KRAS-G12C inhibitor and indicate in the discussion that improves two of the limitations of sotorasib and adagrasib, the high-grade toxicity and the target occupancy. The clinical data presented previously nicely shows that olomorasib is well tolerated and does not present high-grade liver toxicities, which should facilitate the development of combinations strategies. In the discussion the authors also indicate that the target occupancy is better than adagrasib and sotorasib and cite two articles to support this data. I have found difficult to find comparable experiments that support this. Do they refer to in vivo or in vitro data? The manuscript shows target occupancy in vitro in Figure 1F but the data in vivo seems to be RAS pathway inhibition.
I believe the characterization of olomorasib done in the manuscript is thorough and very useful considering the current clinical stage of this inhibitor. However, it would be interesting to compare some of the parameters measured with the approved (first generation) KRAS-G12C inhibitors.

Another major comment is that the manuscript seems to test a lot of combinations and they “provide a rational for frontline combination therapies with KRAS inhibitors”. However, I find it difficult to extract clear conclusions from the data presented as they test all the combinations that have been previously proposed and everything seems to work. Can something else be extrapolated from the data presented that would indicate which combinations have more potential? It would be interesting to add an additional message to the data previously published.
It is a bit surprising that in line 517 it says that based on the in vitro data examined the combinatorial efficacy of several drugs and that the cell line used is H358, which do not show synergism with any of the treatments tested (probably because they are very sensitive; Supplemental Table 2), although the in vivo dose used is clearly lower than in Figure 3A.
In Figure 4 (and text) probably it would be better to show first all the combinations with H358 and then specifically discuss the combination with EGFR inhibitors in CRC and the combination with anti-PD1.
Interestingly, except for Aurora kinase, the inhibitors that restored the response to olomorasib in the resistant cells (Figure 6) are not shown in the parental cells. Do they also show synergism in the parental cells or is it something specific of the resistant cells?

Additional comments

The initial in vivo experiments in Figure 3 are done using BID whereas the combinations are done using QD. The authors discuss both schedules in the discussion but in the results section the change of treatment schedule is not indicated and there is no comparison between both schedules.

Some references, especially in the introduction do not seem to match the sentence. Please revise the use of references. For example:

Line 84- the references are about dose single sotorasib and adagrasib treatment, at least a reference of sotorasib with anti-PD(L)1 should be added.
Line 98- Why has the reference 18 been added? It is not a paper discussing possible combinations.
Line 427- reference 26 it is not the reference of the RASless MEFs

Some legends like relevant information. For example:
Figure 1: does not indicate the time of treatment.
Figure 2: some drugs indicate the target and some not. It would be useful to indicate the target for all of them.
Legend 5 does not indicate number of repeats.
Statistical tests used are not indicated.

Some methods are missing. For example:
Methods only indicate the brain orthotopic experiment but not the xenograft models.
Dosage and formulation of in vivo drugs is not shown. Same for which anti-PD1 and anti-PD-L1 antibodies have been used.
There is not information about the RASless MEFs. In fact, it is not clear which cells are used as control in the experiments.
Indicate the gRNAs used to knock-in H1373 cells. Indicate the antibodies used.
Can the two western blot protocols be merged?

The stats of the in vivo experiments in Figures 3 and 4 are not difficult to see. Moreover, I do not believe the stats should be done at one specific time point but comparing the curves using a two-way ANOVA. It could be done until de then of the treatment and indicate this in the legend.

Figures 5G and 5H are repetitive. Move to supplementary.

Reviewer #2

(Remarks to the Author)
The manuscript presents the preclinical characterization of olomorasib, a covalent KRAS-G12C inhibitor currently under clinical investigation. The authors report that olomorasib binds selectively to GDP-bound KRAS-G12C, exhibits potent biochemical and cellular activity, retains efficacy against clinically relevant second-site mutations, and enhances anti-tumor responses when combined with pathway inhibitors, including PD-1/PD-L1 blockade. These findings are potentially impactful, particularly given olomorasib's apparent insensitivity to H95X mutations. However, the manuscript requires substantial revision to meet the standards for publication in Nature Communications.

Major points:

The manuscript must explicitly state that olomorasib targets GDP-bound KRAS-G12C, and this information should be consistently reflected in the title, abstract, results, and structural figures.

The origin of the initial chemical matter is not described; the authors should state whether the hit identification used HTS, fragment screening, etc. Please provide brief details.

Include a quantitative comparison of kinact/Ki values with adagrasib and sotorasib, where available, to contextualize the biochemical potency of olomorasib.

Comparative analysis of second-site KRAS-G12C mutations should include sotorasib, not just adagrasib.

Figure 1B lacks sufficient structural detail; include a zoomed-in figure of the binding pocket with labeled interactions and an appropriately contoured electron density map showing the covalent linkage to C12.

The X-ray crystallographic methods are missing and must be added, including beamline, wavelength, temperature, and refinement details.

Supplemental Table 1 is incomplete: it lacks CC1/2, B-factors for water and ligand, number of atoms, and Ramachandran statistics. The completeness (75.8%) is low and should be justified with CC1/2.
The SPR experiments lack essential details: chip immobilization levels, analyte concentrations, theoretical vs. observed Rmax, and whether equal loading was used across proteins. The buffer product number appears incorrect.

Figure 1C includes 11 concentrations but only shows 10 curves, with reused symbols that impair interpretation. Unique symbols or a K_obs vs. [compound] plot would improve clarity.
Hydrolysis and exchange rates (lines 415–416) are cited without data or references.

Figure 3G (line 508) is incorrectly referenced and does not exist—likely refers to the right panel of 3F.

The claim of low liver toxicity contributing to combinatorial efficacy with PD-1/PD-L1 inhibitors is unsubstantiated—data or citation should be included.

The SPR results might be affected by the different off rates of adagrasib vs olomorasib. Perhaps a better experiment will be to repeat the mass spec assay with different double mutants.

Acronyms are used before being defined; revise to ensure all are expanded at first use.

The manuscript requires significant editing to improve grammar, clarity, and readability throughout.

Minor points:

References are missing for PDB IDs 4L8G and 6XHB. Use uppercase letters for PDB IDs to avoid confusion.

Ensure all figure legends clearly describe what is shown (e.g., identify the blue mesh as 2Fo–Fc and indicate contour level).

Ensure all figure callouts in the text match actual figures.

Version 1:

Reviewer comments:

Reviewer #1

(Remarks to the Author)
The authors have addressed most of my comments. However, I consider that although the legends now indicate for which samples the statistical test was done, they do not give all the relevant information about the statistical analysis performed.

- Legend Figure 3- Indicate that the statical analysis is shown in Supplementary Table 3, similarly to the legend in Figure 3.
- Supplementary Tables 3 and 5. Indicate the statistical test used and the conversion of the stars in the Figure to p-value. It does not fit the standard values. Figure 4 has only * or ** but in Supplementary Table S5 most of the p-values are $p<0.0001$
- I still find confusing the stats in Figure 4, especially panel D. I understand that the colours indicate what it is compared to, but the lines are so mixed that it is difficult to see what they indicate, especially the comparison of the single treatments to the vehicle. There are four lines, only ** and from the data in the table not all the comparisons of single treatment to vehicle were significant. One option could be shown only selected stats and indicate that in the legend, as the rest are indicated in the supplementary table. The other option would be to add the stats in the legend that is in the figure (where it indicates the treatments and colour of the lines).
- Supplementary Tables 5. The continuation document that was in the previous version, showing the stats comparing the different treatments seem to be missing in the document that I downloaded.
- Supplementary Figure 3. Indicated the statistical test used.
- I just found a couple of typos reading the manuscript. Line 150 should say Barbacid. Supplementary Figure 6, second line, should say inhibition.

Reviewer #2

(Remarks to the Author)
Most of the scientific comments have been addressed; however, several reviewer suggestions remain unresolved in the manuscript despite the authors’ claims to the contrary.
For example, the acronym SOC is first used on line 77 but is not defined until line 783.

The manuscript also contains numerous stylistic inconsistencies that appear to result from insufficient attention to detail—for instance, the font mismatch in the legend of Supplementary Figure 1.

Based on scientific merit, I support publication, but the authors or the production team at Nature Communications should correct these inconsistencies to ensure clarity and readability.

Open Access This Peer Review File is licensed under a Creative Commons Attribution 4.0 International License, which permits use, sharing, adaptation, distribution and reproduction in any medium or format, as long as you give appropriate credit to the original author(s) and the source, provide a link to the Creative Commons license, and indicate if changes were made.
In cases where reviewers are anonymous, credit should be given to 'Anonymous Referee' and the source.
The images or other third party material in this Peer Review File are included in the article's Creative Commons license, unless indicated otherwise in a credit line to the material. If material is not included in the article's Creative Commons license and your intended use is not permitted by statutory regulation or exceeds the permitted use, you will need to obtain permission directly from the copyright holder.
To view a copy of this license, visit https://creativecommons.org/licenses/by/4.0/

Reviewer 1:

1) The authors describe olomorasib as a second generation KRAS-G12C inhibitor and indicate in the discussion that improves two of the limitations of sotorasib and adagrasib, the high-grade toxicity and the target occupancy. The clinical data presented previously nicely shows that olomorasib is well tolerated and does not present high-grade liver toxicities, which should facilitate the development of combinations strategies. In the discussion the authors also indicate that the target occupancy is better than adagrasib and sotorasib and cite two articles to support this data. I have found difficult to find comparable experiments that support this. Do they refer to in vivo or in vitro data?

   We appreciate the reviewer's thoughtful feedback and the opportunity to clarify the nature of the data supporting our conclusions regarding olomorasib's target occupancy.
   In the revised manuscript, we have now explicitly distinguished between in vitro and in vivo data supporting olomorasib's superior target occupancy profile:

   In Vitro Evidence: As shown in Figure 1F, olomorasib demonstrates >90% target occupancy in KRASG12C-mutant cell lines during the 24-hour dosing period. This is supported by biochemical assays and mass spectrometry-based chemical proteomics, which confirmed selective engagement of KRASG12C cysteine residues with minimal off-target binding

   In Vivo Evidence: In xenograft and patient-derived xenograft (PDX) models, olomorasib treatment resulted in robust, dose-dependent inhibition of RAS activity, RAS-GTP, and pERK expression. These pharmacodynamic markers are consistent with sustained target engagement in vivo

   While direct in vivo occupancy measurements are technically challenging, the observed downstream pathway inhibition and tumor growth suppression provide strong indirect evidence of high target occupancy.

   Comparative Data: The manuscript now includes a clearer comparison of olomorasib's occupancy (>90%) with published values for adagrasib (<80%) and sotorasib (88%)

   These values are derived from both internal preclinical studies and referenced literature, and are now explicitly cited and contextualized in the discussion.

2) The manuscript shows target occupancy in vitro in Figure 1F but the data in vivo seems to be RAS pathway inhibition. I believe the characterization of olomorasib done in the manuscript is thorough and very useful considering the current clinical stage of this inhibitor. However, it would be interesting to compare some

of the parameters measured with the approved (first generation) KRAS-G12C inhibitors.
We thank the reviewer for the positive feedback on the characterization of olomorasib and for the suggestion to enhance the comparative context with first-generation KRAS-G12C inhibitors.

In response, we have expanded the discussion to more explicitly compare olomorasib with adagrasib and sotorasib across several key pharmacologic and pharmacodynamic parameters:

Target Occupancy: Olomorasib achieves >90% target occupancy during the 24-hour dosing period, which exceeds the reported occupancy of adagrasib (<80%) and sotorasib (88%) under similar conditions. This is supported by both *in vitro* biochemical assays and *in vivo* pharmacodynamic markers such as RAS-GTP and pERK suppression in xenograft and PDX models. Previously mentioned in the discussion. To this point, we have also updated the Supplemental Figures to include our internal data, demonstrating overall better target occupancy of olomorasib compared to first-generation KRAS-G12C inhibitors.

Toxicity Profile: Unlike adagrasib and sotorasib, which are associated with high-grade liver toxicities that limit combination strategies, olomorasib has demonstrated a favorable safety profile in early clinical trials, with no high-grade liver toxicity observed. This improved tolerability supports its use in combination regimens, including with anti-PD-1/PD-L1 agents. Previously mentioned in the discussion.

Structural and Mechanistic Advantages: Structurally, olomorasib's 8-membered heterocycle constrains the molecule to efficiently deliver the acrylate to C12, resulting in a high-affinity, low-strain covalent bond with $KRAS^{G12C}$. This design contributes to its superior potency and selectivity, as confirmed by mass spectrometry-based proteomics.

Efficacy in Resistant Models: Olomorasib has shown activity against second-site $KRAS^{G12C}$ mutations (e.g., H95Q, H95R, Y96C) that confer resistance to first-generation inhibitors. This suggests a broader therapeutic window and potential utility in patients with acquired resistance. Previously mentioned in the discussion.

Combination Potential: In syngeneic mouse models, olomorasib combined with anti-PD-1 or PD-L1 antibodies led to enhanced tumor regression without added toxicity. This contrasts with the limited combinability of sotorasib and adagrasib due to overlapping toxicities. Previously mentioned in the discussion.

3) Another major comment is that the manuscript seems to test a lot of combinations and they "provide a rational for frontline combination therapies with

KRAS inhibitors". However, I find it difficult to extract clear conclusions from the data presented as they test all the combinations that have been previously proposed and everything seems to work. Can something else be extrapolated from the data presented that would indicate which combinations have more potential? It would be interesting to add an additional message to the data previously published.

We thank the reviewer for this insightful comment. We agree that while the manuscript explores a broad range of rational combination strategies, it is important to distill a more focused message that highlights the most promising and clinically actionable combinations. In response, we have revised the discussion to emphasize mechanistic rationale, translational alignment, and emerging clinical validation for prioritized combinations.

Mechanism-Based Prioritization: Among the combinations tested, those targeting upstream (e.g., EGFR via cetuximab) and downstream (e.g., ERK, CDK4/6) RAS pathway nodes demonstrated the most consistent and synergistic effects in both in vitro and in vivo models. These combinations were selected based on known mechanisms of adaptive resistance to KRASG12C inhibition, including feedback reactivation of EGFR signaling and MAPK pathway rebound.

Clinical Relevance of Cetuximab + Olomorasib in Colorectal Cancer: We now highlight the combination of olomorasib with cetuximab as a particularly compelling strategy in $KRAS^{G12C}$-mutant colorectal cancer (CRC). In our preclinical models, this combination showed synergistic activity in vitro and led to tumor regression and delayed growth in vivo. Importantly, this combination is currently under clinical investigation in a Phase 1/2 study (NCT04956640), which has reported promising early results in patients with advanced $KRAS^{G12C}$-mutant CRC. These findings support the translational relevance of our preclinical data and provide a strong rationale for prioritizing this combination in CRC.

Translational Alignment and Safety: Unlike first-generation $KRAS^{G12C}$ inhibitors, olomorasib has not reported high-grade liver toxicity in clinical trials, which enables safer combination with agents like cetuximab and immune checkpoint inhibitors. This improved safety profile enhances the feasibility of combination strategies in both frontline and refractory settings.

Proposed Prioritization Framework: Based on the above, we now propose a tiered prioritization of combinations:

Colorectal Cancer: Olomorasib + cetuximab, supported by mechanistic synergy and ongoing clinical validation.

NSCLC and Pancreatic Cancer: Olomorasib + anti-PD-1/PD-L1, based on robust tumor regression in syngeneic models and early clinical efficacy.

Resistance Settings: Olomorasib + SHP2 or PI3K inhibitors, targeting adaptive reactivation pathways.

We have updated the discussion to include this statement: These findings support a prioritized framework for combination development, with cetuximab + olomorasib in colorectal cancer and pembrolizumab + olomorasib in NSCLC as clinically actionable strategies.

4) It is a bit surprising that in line 517 it says that based on the in vitro data examined the combinatorial efficacy of several drugs and that the cell line used is H358, which do not show synergism with any of the treatments tested (probably because they are very sensitive; Supplemental Table 2), although the in vivo dose used is clearly lower than in Figure 3A.

   We agree with the reviewer's observation. *In vitro*, H358 cells were quite sensitive to olomorasib treatment, which limited our synergism calculations, while *in vivo*, there was a more moderate response.

5) In Figure 4 (and text) probably it would be better to show first all the combinations with H358 and then specifically discuss the combination with EGFR inhibitors in CRC and the combination with anti-PD1.

   We thank the reviewer for this suggestion. Figure 4 has been modified accordingly. H358 cell lines are now Figures 4A and 4B

6) Interestingly, except for Aurora kinase, the inhibitors that restored the response to olomorasib in the resistant cells (Figure 6) are not shown in the parental cells. Do they also show synergism in the parental cells, or is it something specific to the resistant cells?

   We appreciate the reviewer's thoughtful observation. We did observe an increased response to Aurora kinase inhibitors in combination with olomorasib in the parental setting. Notably, this combinatorial effect was more pronounced in the resistant cells, as demonstrated in Figure 6D (black-outlined square). These findings suggest that while some degree of synergy may be present in the parental cells, the enhanced response is more specific and robust in the resistant context.

Additional Comments:

1) The initial in vivo experiments in Figure 3 are done using BID whereas the combinations are done using QD. The authors discuss both schedules in the discussion but in the results section the change of treatment schedule is not indicated and there is no comparison between both schedules.

To assess whether or not there was combination benefit we used QD dosing rather BID. QD was a suboptimal dosing strategy that allowed us to investigate potential therapeutic combination benefit with olomorasib, given the single-agent efficacy we observed.

2) Some references, especially in the introduction do not seem to match the sentence. Please revise the use of references. For example:
Line 84- the references are about dose single sotorasib and adagrasib treatment, at least a reference of sotorasib with anti-PD(L)1 should be added.
This has been updated accordingly.
Line 98- Why has the reference 18 been added? It is not a paper discussing possible combinations.
Thank you – this has been updated accordingly.
Line 427- reference 26 is not the reference of the RASless MEFs
Thank you - A new reference has been updated accordingly.

3) Some legends like relevant information. For example:
Figure 1: does not indicate the time of treatment.
This has been updated accordingly
Figure 2: Some drugs indicate the target, and some do not. It would be useful to indicate the target for all of them.
This has been updated accordingly
Legend 5 does not indicate a number of repeats.
Statistical tests used are not indicated.

4) Some methods are missing. For example:
Methods only indicate the brain orthotopic experiment but not the xenograft models.

We apologize for this oversight. The methods have been updated accordingly.

The dosage and formulation of in vivo drugs are not shown. Same for which anti-PD1 and anti-PD-L1 antibodies have been used.

Materials and methods have been updated accordingly.

There is no information about the RASless MEFs. In fact, it is not clear which cells are used as controls in the experiments.

Thank you for bringing this to our attention. A proper reference has been added for the RASless MEFs.

Indicate the gRNAs used to knock-in H1373 cells. Indicate the antibodies used. Can the two Western blot protocols be merged?

The **gRNA sequence** used was**:** AGACTATCACCATTAAGGT;

- H95R KI Donor (mutation: CAT → ACG)
- Y96C KI Donor (mutation: TAT → TGC)
- Y96D KI Donor (mutation: TAT → GAT)
- H95D KI Donor (mutation: CAT → GAT)

5) The stats of the in vivo experiments in Figures 3 and 4 are difficult to see. Moreover, I do not believe the stats should be done at one specific time point but comparing the curves using a two-way ANOVA. It could be done until the end of the treatment and indicate this in the legend.

   We have indicated that Statistical analysis was performed at the end of the treatment period.

6) Figures 5G and 5H are repetitive. Move to supplementary.

   Figure 5H has been moved to Supplemental Figures (Supplemental Figure 6B)

Reviewer 2:

1) The manuscript must explicitly state that olomorasib targets GDP-bound KRAS-G12C, and this information should be consistently reflected in the title, abstract, results, and structural figures.

   The Title, Abstract and relevant Results have been updated accordingly.

2) The origin of the initial chemical matter is not described; the authors should state whether the hit identification used HTS, fragment screening, etc. Please provide brief details.

   We thank the reviewer for this insightful comment. The initial chemical matter was derived from a structure-based drug design approach, previously mentioned in the manuscript, leveraging a scaffold identified in a previous internal effort. This scaffold originated from the first-generation Lilly $KRAS^{G12C}$ inhibitor program. For additional details, we have now included a reference to the relevant patent (WO2020081282 - KRAS G12C INHIBITORS) in the revised manuscript.

3) Include a quantitative comparison of kinact/Ki values with adagrasib and sotorasib, where available, to contextualize the biochemical potency of olomorasib.

   We appreciate the reviewer's suggestion and have now included a quantitative comparison of the biochemical potency of olomorasib relative to other $KRAS^{G12C}$ inhibitors. Specifically, olomorasib demonstrates a significantly enhanced covalent binding efficiency, with a >10-fold improvement in kinact/Ki compared to adagrasib and sotorasib. This improvement is attributed to olomorasib's high-affinity interaction with $KRAS^{G12C}$, which enhances both binding affinity (Ki) and

conformational rigidity for covalent kinetics (kinact). As a result, olomorasib achieves >90% target occupancy over a 24-hour dosing period, outperforming adagrasib (<80%) and sotorasib (88%). These biochemical advantages translate into robust, dose-dependent inhibition of RAS activity, RAS-GTP, and pERK expression, along with consistent suppression of RAS-mediated gene expression in $KRAS^{G12C}$-mutant lung and pancreatic cancer models.

The manuscript has been updated accordingly

4) Comparative analysis of second-site KRAS-G12C mutations should include sotorasib, not just adagrasib.

   We appreciate the reviewer's suggestion to broaden the comparative analysis of second-site $KRAS^{G12C}$ mutations. In response, we have updated Supplemental Figure 8 and the corresponding Results/Discussion section to include sotorasib treatment alongside adagrasib.

5) Figure 1B lacks sufficient structural detail; include a zoomed-in figure of the binding pocket with labeled interactions and an appropriately contoured electron density map showing the covalent linkage to C12

   Figure 1B has been updated to include the zoomed-in figure.

6) The X-ray crystallographic methods are missing and must be added, including beamline, wavelength, temperature, and refinement details.

   The methods have been updated accordingly.

7) Supplemental Table 1 is incomplete: it lacks CC1/2, B-factors for water and ligand, number of atoms, and Ramachandran statistics. The completeness (75.8%) is low and should be justified with CC1/2.

   Supplemental Table 1 has been updated accordingly.

8) The SPR experiments lack essential details: chip immobilization levels, analyte concentrations, theoretical vs. observed Rmax, and whether equal loading was used across proteins. The buffer product number appears incorrect.

   The materials and methods have been updated to include this information

9) Figure 1C includes 11 concentrations but only shows 10 curves, with reused symbols that impair interpretation. Unique symbols or a K_obs vs. [compound] plot would improve clarity.
   Hydrolysis and exchange rates (lines 415–416) are cited without data or references.

We thank the reviewer for their careful evaluation of Figure 1C. We confirm that 11 concentrations were tested and plotted; however, the final two curves (corresponding to the highest concentrations) overlap due to similar binding kinetics, which may have made them difficult to distinguish visually.

The manuscript and references have been updated accordingly.

10) Figure 3G (line 508) is incorrectly referenced and does not exist—likely refers to the right panel of 3F.

We thank the reviewers for bringing this to our attention. The reference to Figure 3G has been updated accordingly (Figure 3F).

11) The claim of low liver toxicity contributing to combinatorial efficacy with PD-1/PD-L1 inhibitors is unsubstantiated—data or citation should be included.

We thank the reviewers for their feedback. With regard to Olomorasib liver toxicity, we reference (ref 33): doi 10.1158/1538-7445.Am2023-ct028

12) The SPR results might be affected by the different off rates of adagrasib vs olomorasib. Perhaps a better experiment will be to repeat the mass spec assay with different double mutants.

We appreciate the reviewer's thoughtful suggestion regarding the potential influence of differing off-rates between adagrasib and olomorasib on the SPR results. We agree that kinetic differences, particularly in dissociation rates, can impact interpretation of binding data. In our current study, we addressed this by fitting the SPR sensorgrams using a 1:1 Langmuir binding model and confirming that the observed R_{max} values aligned with theoretical expectations, supporting the robustness of the binding interactions observed.

While we recognize that mass spectrometry-based occupancy assays using additional KRAS double mutants could provide further mechanistic insight, our current dataset was designed to focus on comparative binding under standardized conditions. All proteins were immobilized at equal levels, and analyte concentrations were carefully titrated to ensure consistent interpretation across compounds. Given the scope of this study and the strong agreement between SPR and biochemical occupancy data, we believe the current results sufficiently support the conclusions drawn.

13) Acronyms are used before being defined; revise to ensure all are expanded at first use.

We thank the reviewers for bringing this to our attention. We have modified the text accordingly.

14) The manuscript requires significant editing to improve grammar, clarity, and readability throughout

The text has been revised accordingly.

Minor points:

1) References are missing for PDB IDs 4L8G and 6XHB. Use uppercase letters for PDB IDs to avoid confusion.

   References have been added accordingly

2) Ensure all figure legends clearly describe what is shown (e.g., identify the blue mesh as 2Fo–Fc and indicate contour level).

   The Figure legends have been updated accordingly.

3) Ensure all figure callouts in the text match actual figures.

   Thank you for pointing this out. We have carefully reviewed the manuscript and ensured that all figure callouts in the text now accurately correspond to the correct figure panels. Any inconsistencies have been corrected for clarity.

REVIEWERS' COMMENTS

Reviewer #1 (Remarks to the Author):

The authors have addressed most of my comments. However, I consider that although the legends now indicate for which samples the statistical test was done, they do not give all the relevant information about the statistical analysis performed.

1. Legend Figure 3- Indicate that the statical analysis is shown in Supplementary Table 3, similarly to the legend in Figure 3. **This has been updated accordingly**

2. Supplementary Tables 3 and 5. Indicate the statistical test used and the conversion of the stars in the Figure to p-value. It does not fit the standard values. Figure 4 has only * or ** but in Supplementary Table S5 most of the p-values are $p<0.0001$. **This has been updated accordingly**

3. I still find confusing the stats in Figure 4, especially panel D. I understand that the colours indicate what it is compared to, but the lines are so mixed that it is difficult to see what they indicate, especially the comparison of the single treatments to the vehicle. There are four lines, only ** and from the data in the table not all the comparisons of single treatment to vehicle were significant. One option could be shown only selected stats and indicate that in the legend, as the rest are indicated in the supplementary table. The other option would be to add the stats in the legend that is in the figure (where it indicates the treatments and colour of the lines).

For clarity statistical analysis from Figure 4D has been removed, per the reviewers' recommendation. The analysis is presented in Supplementary Tables 4 and 5, respectively, and indicated in the figure legend.

4. Supplementary Tables 5. The continuation document that was in the previous version, showing the stats comparing the different treatments seem to be missing in the document that I downloaded.

To streamline interpretation, only the most relevant statistical analyses comparing key treatment combinations have been retained in this version.

5. Supplementary Figure 3. Indicated the statistical test used.

This has been updated accordingly.

6. I just found a couple of typos reading the manuscript. Line 150 should say Barbacid. Supplementary Figure 6, second line, should say inhibition.

1) Updated accordingly, thank you for this note. 2) Updated accordingly.

Reviewer #2 (Remarks to the Author):

Most of the scientific comments have been addressed; however, several reviewer suggestions remain unresolved in the manuscript despite the authors' claims to the contrary.

1. For example, the acronym SOC is first used on line 77 but is not defined until line 783.

This has been updated accordingly.

2. The manuscript also contains numerous stylistic inconsistencies that appear to result from insufficient attention to detail—for instance, the font mismatch in the legend of Supplementary Figure 1.

This has been updated accordingly.

Based on scientific merit, I support publication, but the authors or the production team at Nature Communications should correct these inconsistencies to ensure clarity and readability.
